# Tetraploids expanded beyond the mountain niche of their diploid ancestors in the mixed-ploidy grass *Festuca amethystina* L.

**DOI:** 10.1038/s41598-021-97767-6

**Published:** 2021-09-21

**Authors:** Marcin Kiedrzyński, Katarzyna M. Zielińska, Iwona Jedrzejczyk, Edyta Kiedrzyńska, Przemysław P. Tomczyk, Agnieszka Rewicz, Monika Rewers, Adrian Indreica, Iryna Bednarska, Vladimir Stupar, Jan Roleček, Petr Šmarda

**Affiliations:** 1 Department of Biogeography, Paleoecology and Nature Conservation, Faculty of Biology and Environmental Protection, University of Lodz, Lodz, Poland; 2 Department of Geobotany and Plant Ecology, Faculty of Biology and Environmental Protection, University of Lodz, Lodz, Poland; 3 Laboratory of Molecular Biology and Cytometry, Department of Agricultural Biotechnology, Bydgoszcz University of Science and Technology, Bydgoszcz, Poland; 4 European Regional Centre for Ecohydrology of the Polish Academy of Sciences, Lodz, Poland; 5 UNESCO Chair on Ecohydrology and Applied Ecology, Faculty of Biology and Environmental Protection, University of Lodz, Lodz, Poland; 6 Department of Silviculture, Transilvania University of Brasov , Brasov, Romania; 7 Department of Nature Ecosystems Protection, Institute of Ecology of the Carpathians NASU, Lviv, Ukraine; 8 Faculty of Forestry, University of Banja Luka, Banja Luka, Bosnia and Herzegovina; 9 Department of Botany and Zoology, Faculty of Science, Masaryk University, Brno, Czech Republic; 10 Department of Paleoecology, Institute of Botany, Czech Academy of Sciences, Brno, Czech Republic

**Keywords:** Biogeography, Evolutionary ecology, Biodiversity

## Abstract

One promising area in understanding the responses of plants to ongoing global climate change is the adaptative effect of polyploidy. This work examines whether there is a coupling between the distribution of cytotypes and their biogeographical niche, and how different niches will affect their potential range. The study uses a range of techniques including flow cytometry, gradient and niche analysis, as well as distribution modelling. In addition, climatic, edaphic and habitat data was used to analyse environmental patterns and potential ranges of cytotypes in the first wide-range study of *Festuca amethystina*—a mixed-ploidy mountain grass. The populations were found to be ploidy homogeneous and demonstrate a parapatric pattern of cytotype distribution. Potential contact zones have been identified. The tetraploids have a geographically broader distribution than diploids; they also tend to occur at lower altitudes and grow in more diverse climates, geological units and habitats. Moreover, tetraploids have a more extensive potential range, being six-fold larger than diploids. Montane pine forests were found to be a focal environment suitable for both cytotypes, which has a central place in the environmental space of the whole species. Our findings present polyploidy as a visible driver of geographical, ecological and adaptive variation within the species.

## Introduction

Anthropogenic climate change is predicted to result in dramatic effects on ecosystems across the planet^1^, and there is a great need to predict their influence on species and communities which respond to ongoing stressful conditions^2^. In the case of plants, it is important to assess the role of plasticity and evolutionary adaptations in shaping responses such as buffering, adapting, or moving opportunities in response to climate change^3^. In this regard, one especially promising area of research is the adaptative effect of polyploidy, i.e. the multiplication of an entire chromosome set (genome), which is an important catalyst of ecological diversification in plants^4,5^. Polyploidy-related changes in genomes can lead to changes in physiology, metabolism and morphology, and prevent significant drops in fitness during stressful environmental conditions^6^. Despite progress in understanding the genetic and genomic effects of polyploidy, their ecological consequences remain poorly understood^7,8^. Such studies have considerable social and environmental significance, particularly when considering how many polyploid species are ecosystem dominant and economically important^5^.

In natural conditions, neopolyploid lineages are much more likely to become established if the ecological specialization is able to evolve^9,10^. The most appropriate models for studies of the adaptative effects of polyploidy are those based on mixed-ploidy species^11^. In such complexes, the knowledge of the distribution of cytotypes within species range and its ecological niches may clarify the processes that influence polyploid establishment and adaptation. If polyploids have broader ecological amplitudes and lower inbreeding depression, and are more selfing, they may be better colonizers^4^. Under these circumstances, local allopatry may eventually evolve to regional allopatry and have a biogeographical effect, affecting the distribution pattern of cytotypes and their various adaptations^12^. Hence, polyploidy is considered an important genetic determinant of species range^13^, which in the case of mixed-ploidy species is determined by cytotype distribution^11^.

Indeed, polyploidy may well exert a significant influence on the ecological or geographic range of a species^14^. However, while some polyploids have a broader range^15–17^, this is certainly not a general trend and many species demonstrate the opposite relationship^13,18^. Hence, the question of what drives the shifts in range between diploid and polyploid relatives remains open.

The development of flow cytometry hastened a new wave of environmental studies on polyploids, as the method allows fast estimation of ploidy level^19^. Such knowledge of ploidy levels in populations containing mixed-ploidy species allows further analysis along geographical and altitudinal lines and a clearer description of the ecological niches of cytotypes^20^.

We analyze geographical patterns and potential range within a mixed-ploidy species, based on the example of tufted fescue: *Festuca amethystina* L. (Poaceae). The evolution of the grasses has been accompanied by frequent and repeated genome size gains and losses: about 60% of grass species are classified as polyploids^21–23^, and polyploid grasses dominate in the major grassland ecosystems of the planet^24^. *Festuca* L. is the most species-rich genus of the grasses, with considerable diversification and worldwide distribution, and about 70% of those taxa are thought to be polyploid^25^. Within the fescues, the fine-leaved clade to which *F. amethystina* belongs is also a rapidly-evolving group^26,27^. Its members have xero-cryophytic and light-demanding adaptations, which allow dominance in grassland vegetation from alpine to steppe biomes and their occurrence in open forests.

*F. amethystina* is distributed throughout Central and South-Eastern Europe^28^. Its range, connected with mountain chains, is strongly disjunct and, in many regions, is represented only by residual and relict patches suggesting habitat shortage since the LGM, i.e. the Last Glacial Maximum^29,30^. This intricate geographical pattern, potentially driven by complex glaciation history in the region, may also be associated with its distribution of diploid (2x = 14)^31^ and tetraploid forms (4x = 28)^32,33^. The species is an excellent example of a mountain plant exposed to climate change^34^. In addition, recent global models predict that montane species such as *F. amethystina* will demonstrate complicated and elevation-dependent responses to climate change^35^; furthermore, as this particular mixed-ploidy species has been found to grow in a wide range of habitats, from subalpine to lowland areas, it is an especially promising subject whose analysis can yield a deeper, intraspecific understanding of the response of mountain plants to climate change.

Recently, extensive ploidy variation was found across different parts of the *F. amethystina* range^27,36–38^. Unfortunately, these findings were not sufficient for complex biogeographical analysis, and data were missing from some parts of the distribution range. To address these issues, the present study employs an improved method of population sampling and includes an examination of ploidy levels in populations from the whole geographical range. Therefore, this is the first range-wide study for *F. amethystina*.

This work examines whether a coupling exists between the distribution of cytotypes and their biogeographical niche, and how different niches affect their potential range. The study uses various techniques including flow cytometry, altitudinal and environmental gradients and niche analysis, as well as distribution modelling. It analyses the climatic, altitudinal, habitat and geological preferences of both the diploid and tetraploid forms, and compares the distribution of both cytotypes with those predicted using the machine-learning Maxent algorithm^39^. Our study serves as an example for the analysis of factors driving cytotype distribution by providing analysis in geographical and environmental space.

## Results

### Cytogenetic analysis

**Figure 1:**
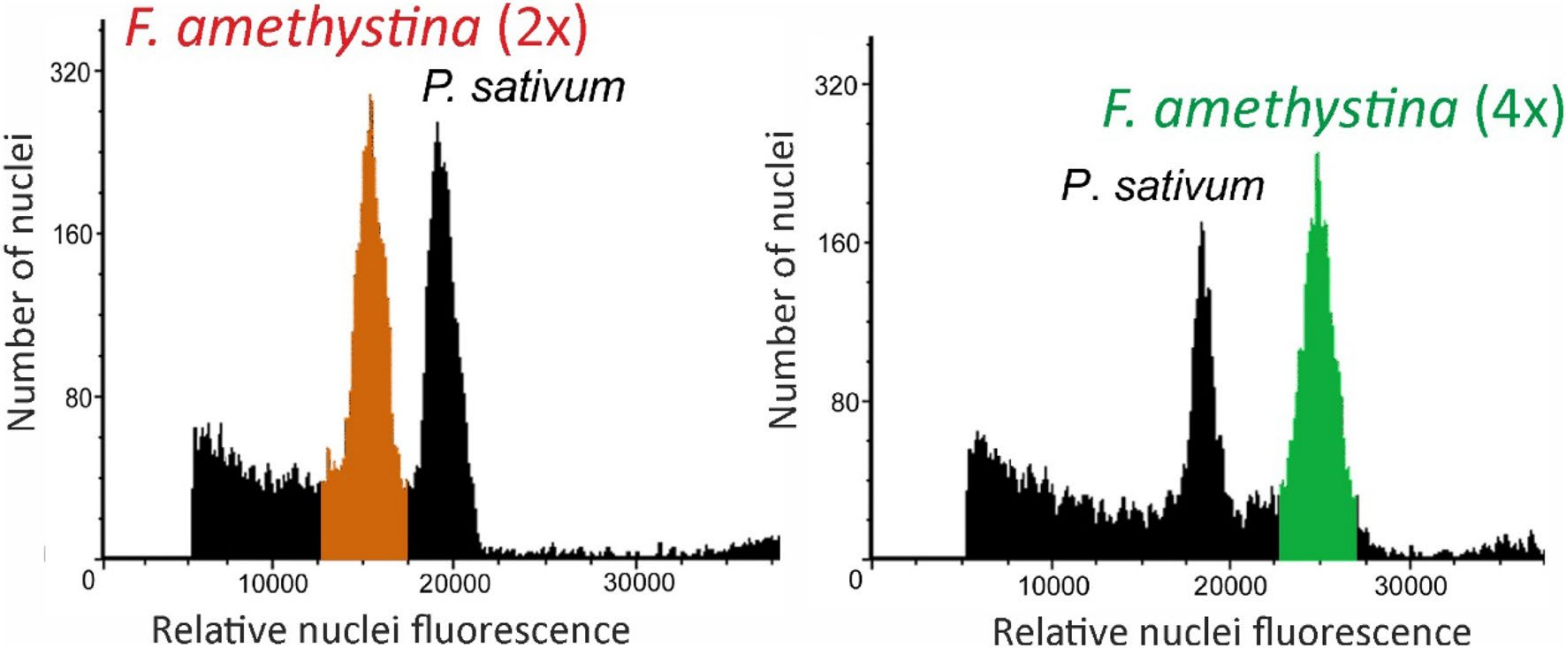
Histograms of relative nuclei fluorescence obtained after FCM analysis of *Pisum sativum* cv. ‘Set’ (internal standard) and example accession of studied cytotypes; representants are: *F. amethystina* (2x)—Corongis, E. Carpathians, Romania; and *F. amethystina* (4x)—Vlašić, Dinaric Mts., Bosnia and Herzegovina.

Ploidy level was estimated in 436 plants from 59 populations of *F. amethystina* (example histograms are shown in Fig. 1). All studied populations of *F. amethystina* were ploidy homogeneous, i.e. diploid or tetraploid (Supplementary Table S1).

### The patterns of geographic occurrence

**Figure 2:**
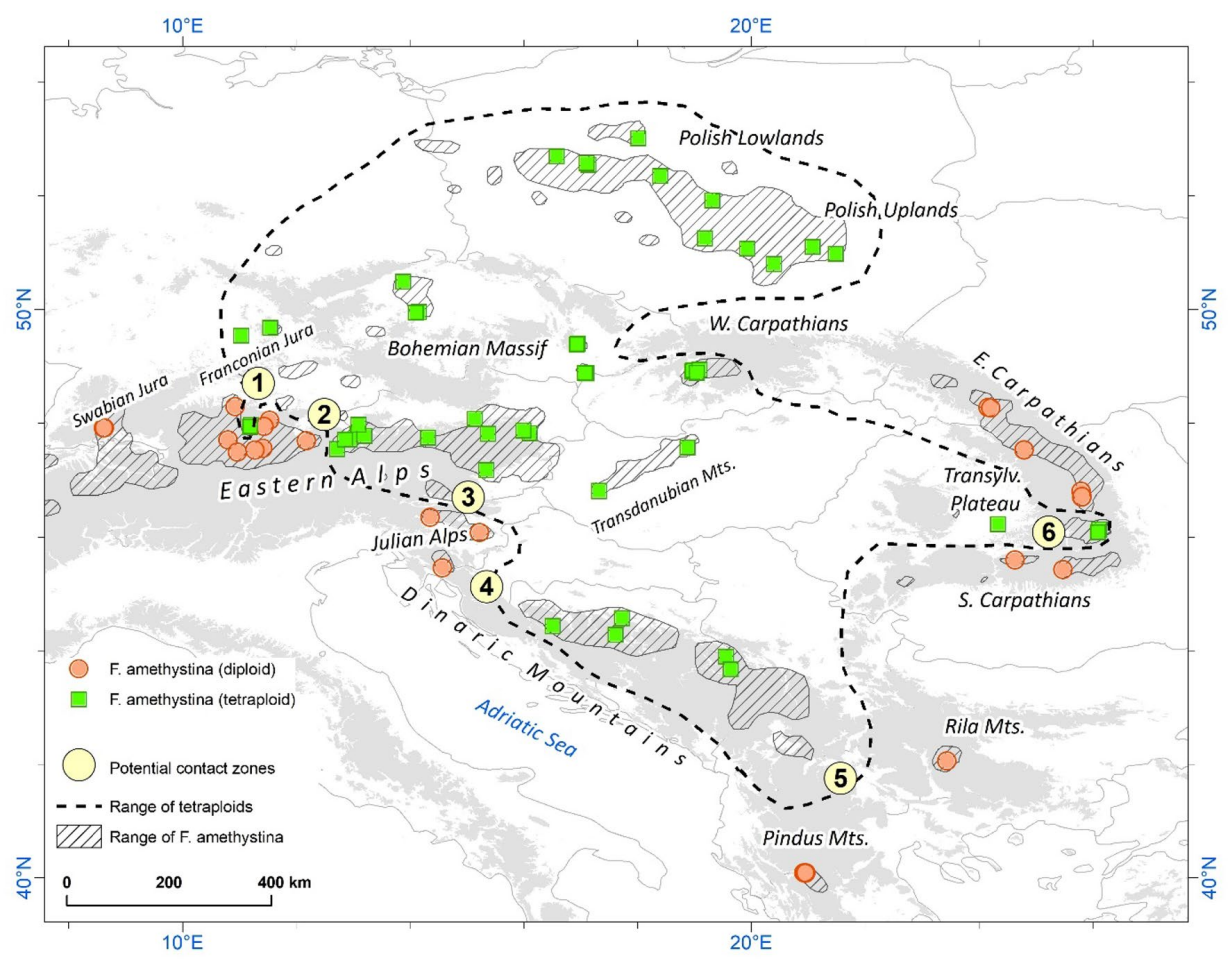
Distribution of diploid and tetraploid cytotypes of *F. amethystina*. Ploidy level in populations was estimated based on flow cytometry analysis (59 populations) or adapted from literature (11 populations). All populations were found to exhibit homogeneous ploidy. The grey areas indicate an altitude above 500 m. The distribution range of *F. amethystina* is an updated version of that identified by Kiedrzyński et al.^30^. The numbered potential contact zones between cytotypes are described in Supplementary Table S5.

The pattern of distribution of diploid and tetraploid *F. amethystina* is parapatric: a general allopatric distribution with a few areas with closer occurrence, which could act as potential contact zones (Fig. 2). In general, the central part of the species range is occupied by tetraploids, and the marginal area by diploids. The most visible exception is the presence of tetraploid populations on the northern border of the species range, e.g., in the Polish Lowlands. Six areas were found to be potential contact zones between cytotypes of *F. amethystina* (marked in Fig. 2 and described in detail in Supplementary Table S5). It can also be seen that diploids of *F. amethystina* are associated with mountain ranges, such as the Alps, Carpathians and mountains of the Balkan Peninsula, while tetraploids occur both in the mountains and lowland areas, particularly the Polish Lowlands (Fig. 2).

### Altitudinal-climatic gradients

**Figure 3:**
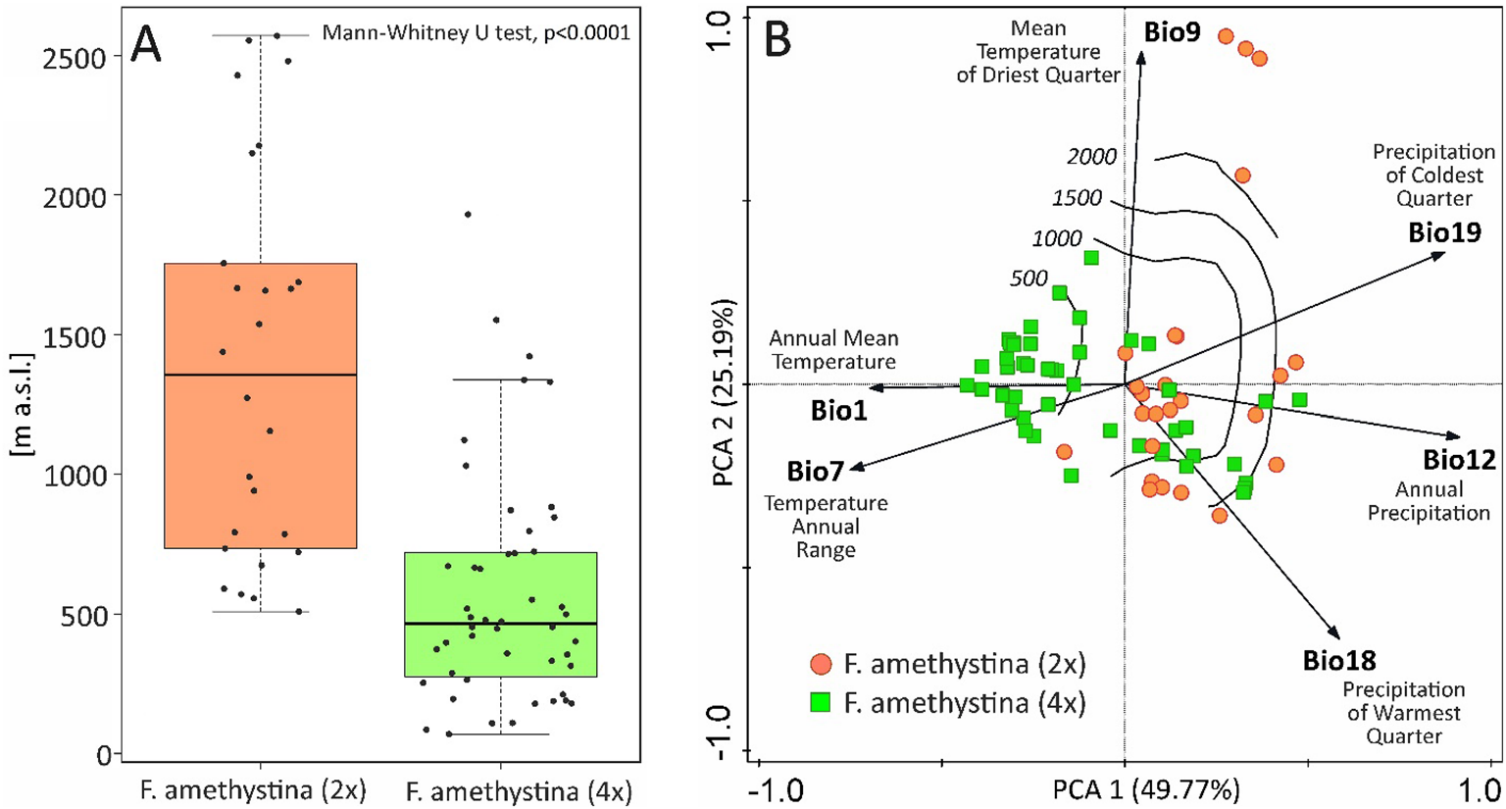
Localities of *F. amethystina* cytotypes according to altitude (**A**) and in PCA multidimensional climatic space (**B**). Boxplots in panel (**A**): the solid lines represent the median, the ends of the boxes indicate upper and lower quartiles, the whiskers show the highest and lowest value excluding outliers, the black dots show the distribution of all localities. Panel (**B**): contours with values corresponding to the altitude [m] estimated by Generalized Additive Model (GAM) according to the PCA axes.

Our results indicate that diploids tend to prefer higher altitudes than tetraploids, and they are not found in altitudes below 500 m. In contrast, tetraploids tend to occur at lower elevations and are not found in the high montane elevations above 2000 m. Both cytotypes are found in middle montane elevations (Fig. 3A, Supplementary Table S3).

**Figure 4:**
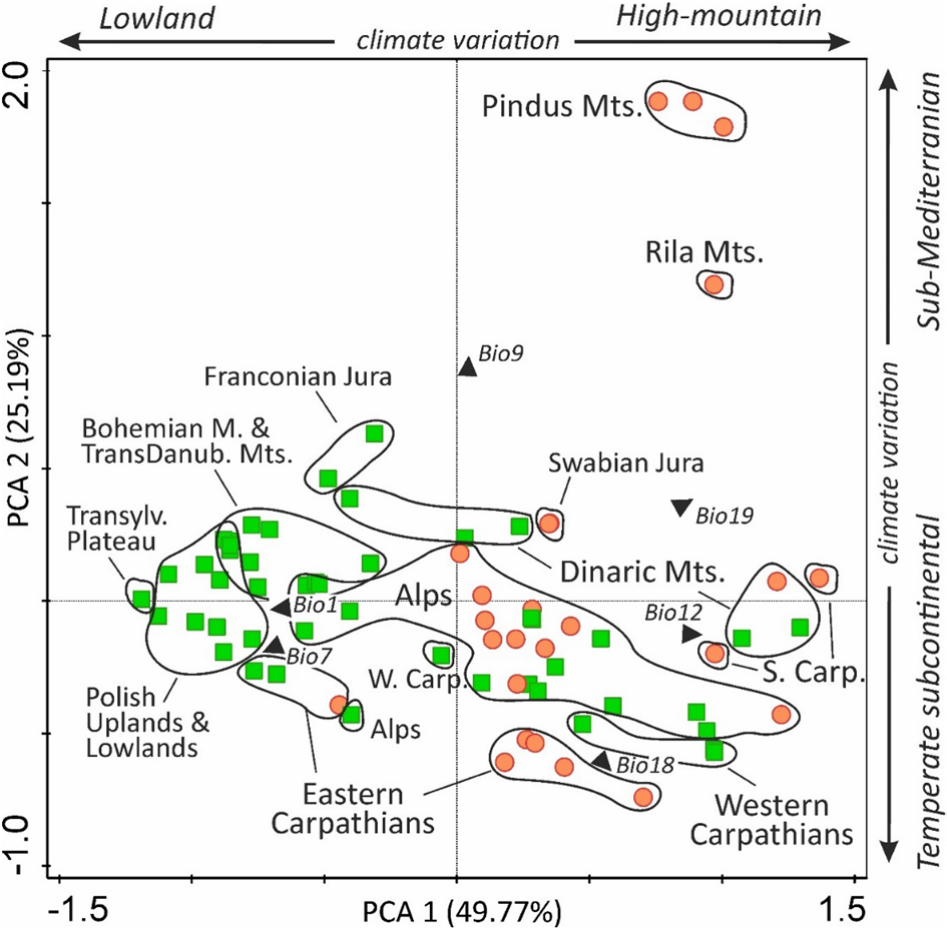
Assignment of the localities of *F. amethystina* to geographical regions concerning PCA climatic space. Red circles—*F. amethystina* (2x), green squares—*F. amethystina* (4x); Black arrowheads indicate the centroids and directions of the climatic factors: Bio1—Annual Mean Temperature, Bio7—Annual Temperature Range, Bio9—Mean Temperature of the Driest Quarter, Bio12—Annual Precipitation, Bio18—Precipitation of the Warmest Quarter, and Bio19—Precipitation of the Coldest Quarter.

Both cytotypes occur in climates that have a relatively higher level of precipitation, both annually and in the warmest quarter (Fig. 3B), and therefore favour mountain regions and temperate subcontinental climate (Fig. 4). The marginal climates for diploids (high-altitude localities in Pindus Mts., Rila Mts.—see Fig. 4) are characterised by higher temperatures of the driest quarter; this is characteristic of sub-Mediterranean regions with a dry period during the warmer part of the year. Lowland localities of tetraploids are characterised by relatively higher annual temperatures and a higher annual temperature range typical for lowland regions in a temperate subcontinental climate (Figs. 3B, 4).

According to the analysis including Maxent niche modelling, the most influenced predictors in models performed for *F. amethystina* diploids were annual precipitation (Bio12), annual mean temperature (Bio1) and precipitation in the warmest quarter (Bio18) (Table 1), while the most influenced in models performed for *F. amethystina* tetraploids was the mean temperature of the driest quarter (Bio9). In addition, Bio12, for diploids, and Bio9, for tetraploids, achieved above 50% contribution when only climatic predictors were used. When the geology layer was added to models, it was essential as a predictor and even the most influenced variable in the case of tetraploids (47.1%). For diploids, the influence was more evenly distributed between parameters in all tested models (Table 1).

**Table 1:** Percentage contribution of predictors used in Maxent niche modelling in diploids and tetraploids of *Festuca amethystina*. Models with two sets of predictors were used and compared in the analysis: only climatic and climatic + geology (parent material of soil) variables.

Predictors	Diploids		Tetraploids	
	Clim	Clim + Geo	Clim	Clim + Geo
**Temperature**				
Bio1 —Annual Mean Temperature	16.8	18.6	7.2	1.0
Bio7 —Annual Temperature Range	10.2	6.5	23.1	7.4
Bio9 —Mean Temperature of the Driest Quarter	3.0	2.3	52.9	36.6
**Precipitation**				
Bio12—Annual Precipitation	53.4	31.6	1.7	0.0
Bio18—Precipitation of the Warmest Quarter	16.5	15.2	7.5	0.8
Bio19—Precipitation of the Coldest Quarter	0.0	0.0	7.6	7.0
Geology (parent material of soil)	n.a.	25.7	n.a.	47.1

Response curves for the most influenced climatic variables show that the diploids occur most likely in areas with annual precipitation higher than 1000 mm, precipitation of the warmest quarter of around 400 mm and mean annual temperature not higher than 3–5 °C (Supplementary Figs. S2, S3). Tetraploids have a higher optimal temperature range (Bio1 and Bio7), but the most influenced predictor, i.e. the mean temperature of the driest quarter, should be not higher than 0 °C (Supplementary Figs. S2, S3). A clear difference is visible in precipitation of the coldest quarter, where the probability of occurrence of the studied cytotypes is clearly distinguished by values of 100–150 mm (Supplementary Figs. S2, S3). Tetraploids tend to occur in the areas with drier cold periods (winters) while diploids are found in areas with relatively wetter winters.

### Habitat and parent material of soils along with altitude

**Figure 5:**
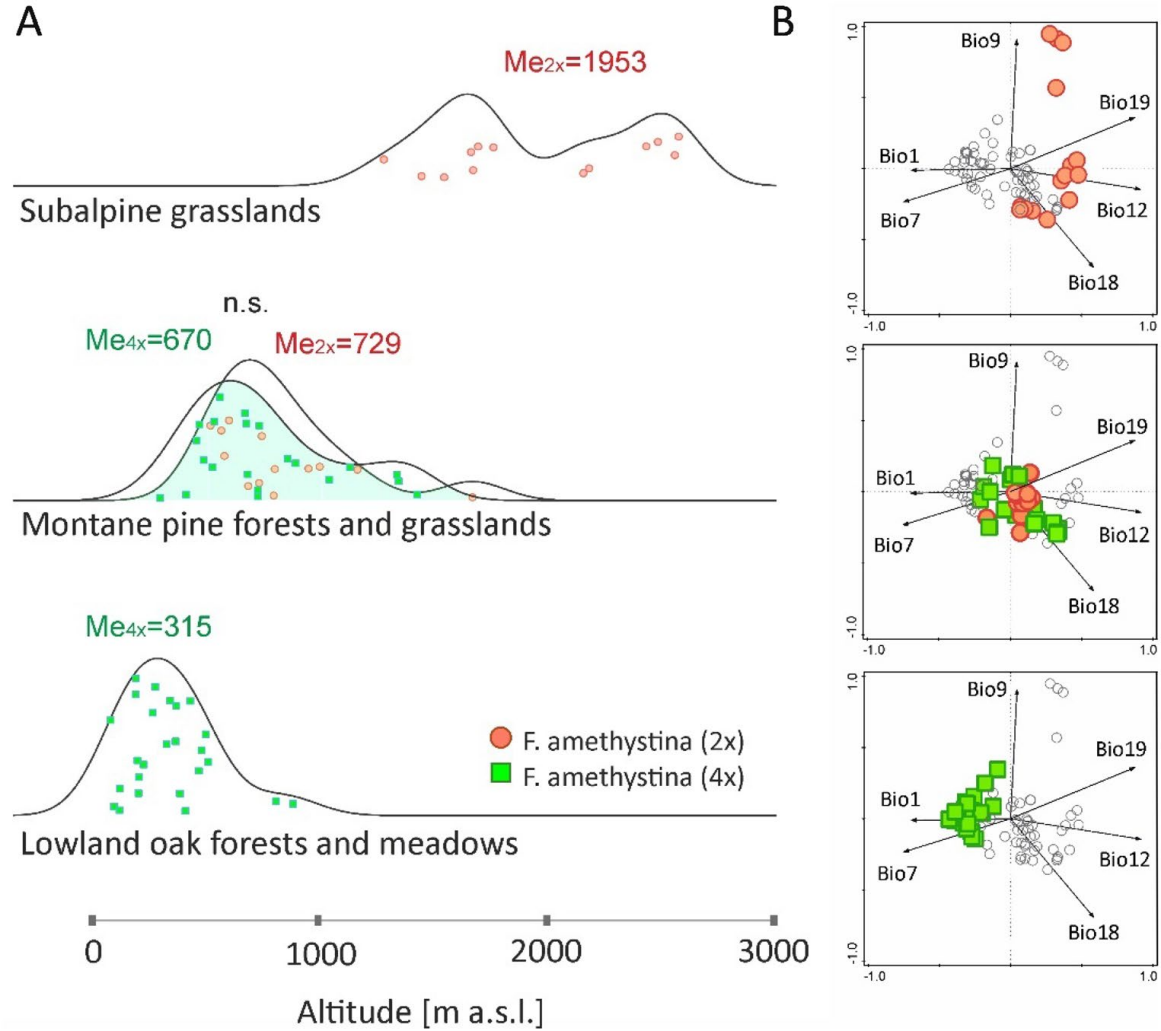
Differentiation of *F. amethystina* cytotypes according to (**A**) the main habitats and altitudinal gradient, visualized as ridgeline plots and (**B**) localities of each habitat in the PCA climatic space with a distinction between cytotypes (extracted from Fig. 3). Me_2x_, Me_4x_—median values of altitude in a given habitat and cytotype, n.s.—non significant statistical differences between medians according to Mann–Whitney *U* test.

The altitudinal gradient of cytotype localities is also reflected in their habitat conditions. Both cytotypes inhabit medium-montane, calcareous pine forests and associated grasslands (Fig. 5A, Supplementary Figs. S4, S6, Table S3); however, difference in altitude between cytotypes within this habitat is not significant. Subalpine calcareous grasslands are almost exclusive to diploids. A single occurrence of tetraploids of *F. amethystina* in subalpine grasslands (Supplementary Figs. S4, S6; not shown in Fig. 5A) was noted in Vlašić Mt and Klekovača Mt (BiH). In contrast, lowland subcontinental oak forests and meadows are exclusive to tetraploids (Fig. 5A), as occasionally are lowland beech forests and grasslands (Supplementary Figs. S4, S6, Table S3, not shown in Fig. 5A).

**Figure 6:**
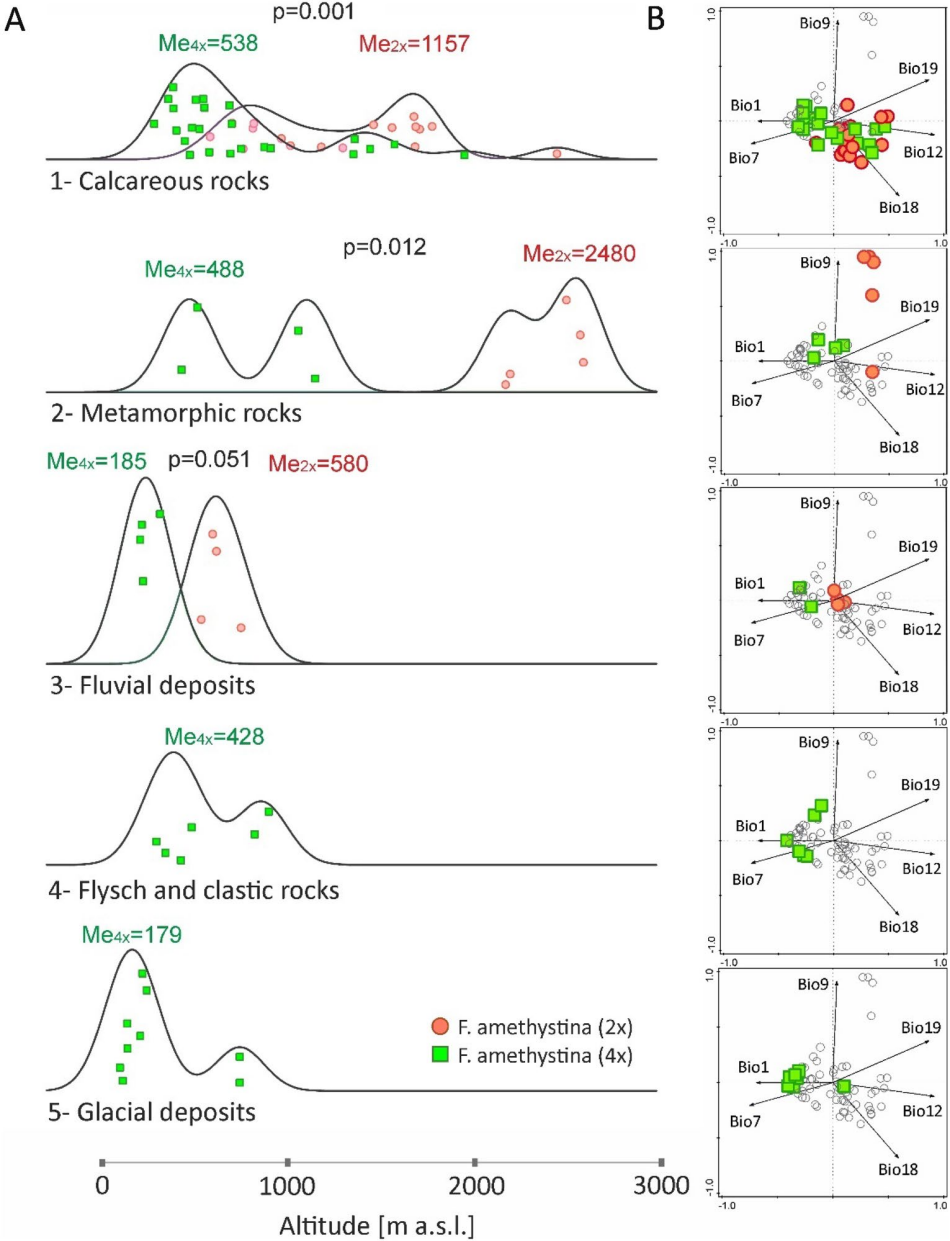
Differentiation of *F. amethystina* cytotypes in (**A**) parent material of soils and altitudinal gradient, visualized as ridgeline plots, and (**B**) localities of each geological unit in the PCA climatic space with a distinction between cytotypes (extracted from Fig. 3). Me_2x_, Me_4x_—median values of altitude in a given type of parent material of soils and cytotype; p-value—significance level of statistical differences between medians according to Mann–Whitney *U* test.

The frequency of cytotypes on different types of soil parent material (geological units) also varies according to altitude (Fig. 6A). Tetraploids of *F. amethystina* demonstrate broader occurrence with regard to geology (units no. 1–5 in Fig. 6A, Supplementary Fig. S5), while diploids have more specific preferences (units 1–3). Flysch and clastic rocks and glacial deposits in lower altitudes are unique to tetraploids. In addition, on metamorphic rocks and fluvial deposits, diploids tend to occur at higher altitudes than tetraploids. A similar pattern is also visible in calcareous rocks, which is the most common parent material  of soils for both cytotypes (Fig. 6A, Supplementary Fig. S7).

The analysis of the habitat, soil parent material and altitudinal gradients (Figs. 5A, 6A, Supplementary Figs. S6, S7), indicates that the focal environment for both *F. amethystina* cytotypes consists of medium-montane pine forest and grasslands on calcareous soils, located in the “central” part of bioclimatic niche of the species (Figs. 5B, 6B). Other habitats and parent materials of soils occupy rather marginal climatic conditions and altitudes.

### Potentially suitable areas

**Figure 7:**
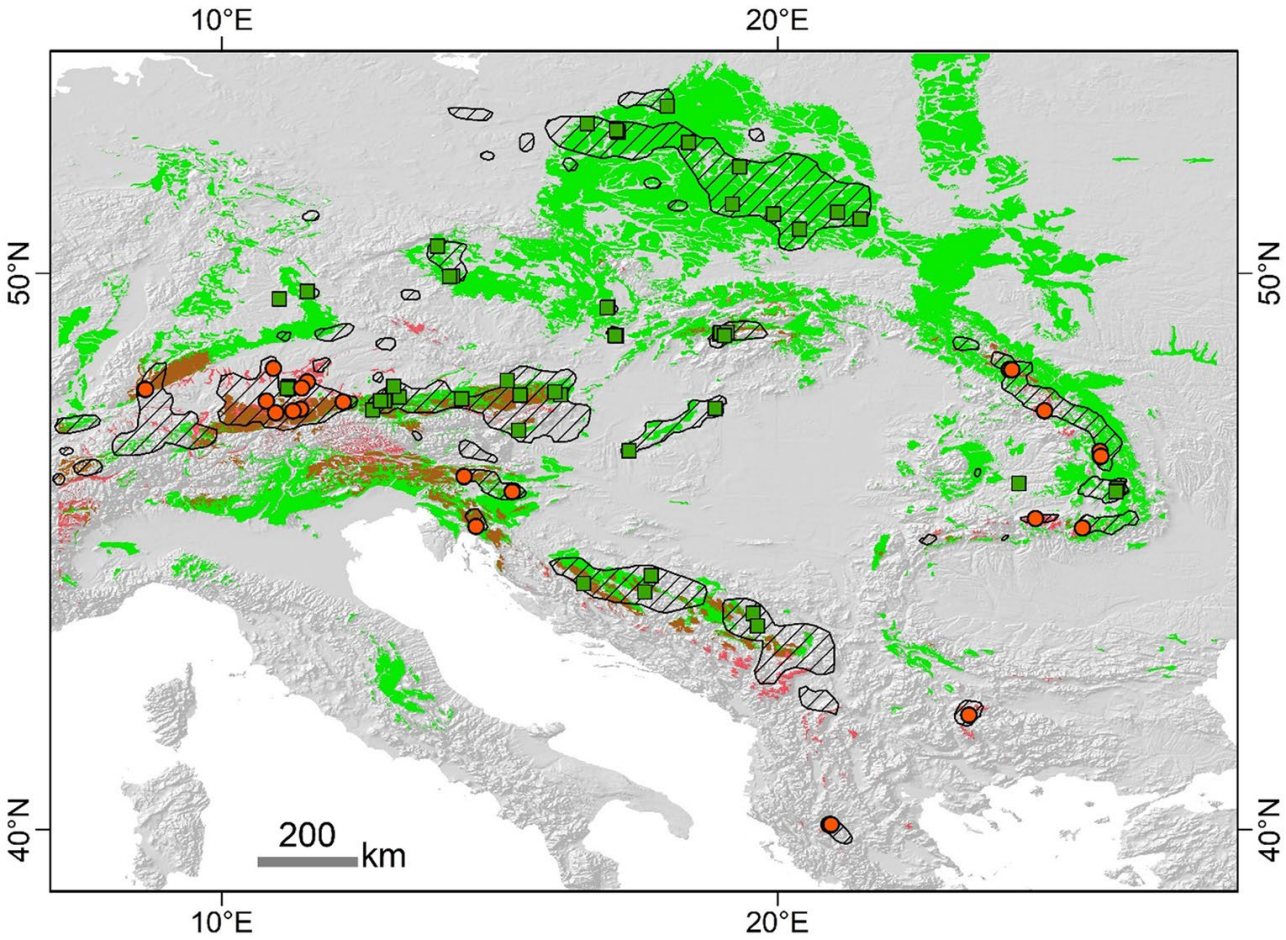
Potentially﻿ suitable areas for the cytotypes of *F. amethystina* according to the projection of the current climate conditions. Maxent models were based on climatic and parent material of soils. The colour areas (red—diploids, green—tetraploids, mixed—both cytotypes) indicates potentially suitable places above the MTSS threshold. Hatching areas—patches of the true range of *F. amethystina* according to Kiedrzyński et al.^30^, updated. Points (red circle—diploids, green squares—tetraploids) indicate locations of cytotypes determined in this study.

The presence–absence prediction maps of suitable areas for the *F. amethystina* cytotypes (Fig. 7) were created based on MTSS thresholds, which for diploids was 0.601 and for tetraploids 0.484.

Areas with potentially suitable conditions for diploids appear to be smaller (MTSS predicted area 81 × 10^3^ km^2^) and concentrated in mountain ranges (Fig. 7). Tetraploids have a more extensive potential range, being six-fold larger (517 × 10^3^ km^2^).

For both cytotypes, models showed areas where they can potentially occur, but they were not found there  (Fig. 7). For example, diploids have potentially suitable areas in the Dinaric Mountains or are more widely distributed in the Eastern and the Southern Alps. Tetraploids tend to be more extensively distributed in the Eastern and Western Carpathians and their northern forelands, as well as in the southern and eastern part of the Alps and the Bohemian Massif.

## Discussion

### Cytotype distribution

Our results indicate that the tetraploids of *Festuca amethystina* have a geographically broader distribution range than the diploids. The differences between the distribution are represented by the presence of tetraploids in lowland localities, especially north of the Carpathians in the Polish territory. However, our modelling of the potential distribution indicated some regions where further examples of both cytotypes may be found by more extensive studies. Polyploids can repeatedly originate in different parts of the species range^8,40^. Hence, the diffuse distribution of tetraploids and adjacent diploid localities indicated in our analysis suggest the relict character of localities or that multiple polyploidization events may have occurred in the past. Moreover, it is also possible that tetraploids from various locations could have arisen from different parents. These scenarios  should be examined in future cytogenetical studies.

*F. amethystina* is perennial, and can persist vegetatively over extended periods of time in discrete habitats; therefore, as demographic processes may be ineffective due to the effect of minority cytotype exclusion, mixed-ploidy populations are possible^41^. However, no such results were obtained in the present study: none of the observed regions demonstrated any sympatric occurrence of *F. amethystina* cytotypes. This suggests that gene flow between cytotypes, if any exists, is currently restricted. This situation could be analogous to the closely-related *F. norica,* where diploids, tetraploids and hexaploids tend to occur in different parts of the Eastern Alps, and probably in most parts of the cytotype ranges populations are ploidy homogeneous^42^.

### Altitude, climatic and habitat preferences of cytotypes

Based on the current state of knowledge, it can be concluded that, in general, polyploids tend to populate higher altitudes than diploids^13^, in terms of the diploid-polyploid ratio and the percentage of polyploids in the mountain flora^43^. However, our present findings indicate a somewhat reversed pattern, where diploids and tetraploids both occur in common medium-montane altitudes, and diploids have rather high-altitude and tetraploids low-altitude localities.

Similar patterns of polyploid occurrence at higher altitudes have also been found for *Atriplex confertifolia*^44^, *Chamerion angustifolium*^45^, *Centaurea jacea*^46^, *Lotus* spp. ^47^ and *Senecio carniolicus*^48^. Some grasses have also been identified where diploids occur at higher elevations than their polyploid descendants, e.g., diploid and tetraploid cytotypes of *Arrhenatherum elatius*^49^ and *Antoxantum alpinum* (2x) and allopolyploid *A. odoratum* (4x) in the *A. odoratum* aggregate.^50^.

Our example represents an interesting pattern of cytogeography, where the parental diploids occupy current mountain habitats, and the polyploids reduce competition to diploids through living in nowadays lowland environments, which can be stressful for mountain plants. Our present findings support the altitudinal segregation of cytotypes with regard to environmental factors: the tetraploids extended their occurrence to general warmer climates, more diverse parent material of soils and habitats. However, in this case, climate seasonality has a visible impact. Tetraploids tend to occur in areas with relatively cold and dry winters; in the studied area characterised by a subcontinental temperate climate.

Similarly, some studies have identified other polyploids that have expanded their niches and ranges beyond those of their progenitors^51–53^. The possibility of occurrence of *F. amethystina* tetraploids in current lowland habitats is connnected with such of cases, when polyploids occupy more temperate and more mesic environments than their diploid ancestors. Such polyploids often have favourable morphological features such as larger leaves, larger stomata and slower growth^54^. Similar ploidy-dependent patterns in plant habit and size have been described for *F. amethystina* in our previous study^37^.

### Tetraploids in lowlands as glacial relicts

The occurrence of mountain species in lowland areas is often considered a relict of previous cold periods. As such, the occurrence of *F. amethystina* in lowland areas has been interpreted as a glacial relict since early biogeographical studies^55^. Our findings indicate that nowadays species-rich lowland oak forests, occasionally also beech forests, or associated meadow and grassland communities harbour only tetraploid populations. Therefore, it should be considered in what sense these lowland populations can be considered as a relict. The ‘glacial relicts’ should then represent the remnants of tetraploid larger ancestral range that occurred during glaciations. The current disrupted range of tetraploids, with many local islands of occurrence, supports this interpretation. However, it appears that this distribution pattern considered on the background of the Pleistocene glacial ranges, is the result of migrations that took place during earlier glaciations, not just the last ice age. These issues may become clearer after examining the phylogeography of the species as a whole.

Considering lowland populations as a relict has further implications for understanding adaptations in such polyploid complexes. Assuming that the habitats in which tetraploids occur play a refugial role, their microclimate should be decoupled from regional trends and demonstrate lower species competition^56^. Tetraploids occurring in lowland forests under the tree canopy can be less exposed to climatic extremes, such as drought and high temperatures^57^. Further experimental studies will aim to discover whether tetraploids have a higher tolerance to lowland conditions than their diploid progenitors, as suggested by our analysis. Alternatively, the occurrence of tetraploids in such regions may be made possible by the specific properties of the refugial habitats rather than the adaptation of plants per se.

Mentioned habitats play a refugial role for many other plants, but are shrinking as a result of the changing anthropogenic regime and, probably, anthropogenic climate change^29,58–60^. As a result, many lowland populations of *F. amethystina* are now considered extinct^61^. If this trend continues, lowland tetraploid populations, which probably harbour extensions of the species ecological niche, are under threat. The extension of species ecological niche demonstrated by lowland populations of tetraploids is in line with the assumption that diploids are older than tetraploids and have a mountain distribution and niche; as such, this *extension* appears to be the ecological constitution of tetraploids, which have apart mountain locations also lowland populations.

## Conclusions

Our data indicate that the cytotype distribution of pure-ploidy populations of *F. amethystina* follows a parapatric pattern. Even populations of cytotypes located in the same region are separated by several dozen kilometres, occur at different altitudes, or exist in different habitats. Tetraploids tend to occur at lower altitudes, on more varied geological units and in more varied habitats than diploids. Our results also identify a focal environment suitable for both cytotypes, which has a central place in the environmental space. 

The range expansion of tetraploids of *F. amethystina* probably occurred as a result of survival in lowland refugia during the Pleistocene climatic oscillations. The studied complex represents an example of polyploid expansion or adaptation to lower elevations than their montane diploid ancestors. In those localities, polyploids currently occur in refugial habitats, where regional climate extremes can be mitigated by topography or vegetation structure. Any direct link between the expansion of polyploids and their adaptations to drier and warmer lowland conditions needs to be determined in further experimental studies.

## Materials and methods

### Plant sampling

The plant material was collected in the field between 2014 and 2019 in different parts of the geographic range of *F. amethystina*. Altogether 55 localities were sampled (Supplementary Table S1). In most cases, to confirm the ploidy of the sampled populations, at least five accessions were collected from plants in different parts of each particular locality. Each accession consisted of several leaves from an individual plant; these were either placed in plastic bags and dried in silica gel, or in paper envelopes and allowed to air dry at room temperature. The geographical coordinates of the centre point of the sampled localities were recorded in the field using a GPS Garmin 60CSX.

Material for four localities was obtained from herbarium sheets deposited in Munich (M) and Lodz (LOD) herbaria (Supplementary Table S1). The herbarium materials were located based on coordinates contained in the source or those determined based on the locality description.

The altitude of the centre point of the localities (Supplementary Table S3), indicated by the GPS coordinates, was determined from Google Earth using our own script.

### Ploidy level determination

The ploidy level of the sampled plants was estimated using flow cytometry (FCM). The nuclear DNA content was measured in the dry leaves of accessions. The samples were prepared for flow cytometric analysis either by using PI according to Rewicz et al.^37^ (Supplementary Table S1) or DAPI, according to Šmarda et al.^27^ (Supplementary Table S1). The present analysis included also FCM measurements of 108 accessions from our previous study mentioned above, and 328 taken for the present one.

In addition, further information on the ploidy level of *F. amethystina* was obtained from previously published studies; however, only data with indicated geographic locations were used: coordinates, locality names or forest complex names which can be precisely localized by topography or distribution of vegetation patches on local maps. Our search revealed twelve additional localities with specified ploidy levels (Supplementary Table S2). Therefore, our analysis included 71 localities with specified ploidy levels.

### Current bioclimatic parameters

Thirty arc second (~ 1 km) resolution raster data was used, incorporating 19 bioclimatic variables from the WorldClim database^62,63^. Firstly, all 19 bioclimatic variables were assigned to each locality from the raster cells. The values were extracted according to the coordinates of localities in the ArcGIS DesktopTM 9.2: Spatial Analyst tools, Extract values to point tool [ESRI Inc. 1999–2008, Redlands, CA, USA].

The procedure of variable selection for analysis assumed that the main climatic variables of precipitation and temperature (Bio1, Bio 12) should be included, as well as some additional values. Variables should not be closely and positively correlated to each other were then identified, as such correlations may impede the interpretation of the principal component and range modelling analyses. We used the correlation matrix to choose the additional climatic values which show seasonality of climate. Correlation matrix was constructed using ‘corrplot’ package in R^64^ (Supplementary Fig. S1). No single threshold of correlation coefficient was used in the procedure. For temperature variables, Pearson’s correlation coefficient can be accepted lower than 0.60 to the main Bio1 variable; for even highly correlated precipitation variables, the correlation coefficient should not exceed 0.85 of the main Bio12 variable. Finally, the distribution of vectors of climatic variables was checked on the Principal Components Analysis (PCA). The selected values should show as far as possible the different directions of vectors in the PCA diagram. Finally, we chose six variables: three climate parameters for temperature (Bio1—annual mean temperature, Bio7—annual temperature range, Bio9—mean temperature of the driest quarter) and three for precipitation (Bio12—annual precipitation, Bio18—precipitation of the warmest quarter, Bio19—precipitation of the coldest quarter).

### General habitats in localities

Each locality was classified as one of the following habitat types: (i) subalpine grasslands, (ii) montane pine forests and grasslands, and (iii) lowland oak forests and meadows, and (iv) lowland beech forests and grasslands. The assignment is based on our field observation/descriptions and supported by published materials (Supplementary Table S3). In the case of materials from herbaria, the habitat was assigned based on the information/descriptions on the herbarium sheets.

### Raster with the parent material of soils

The parent material of the soils (i.e. the geological units) was assigned to localities according to the European Soil Database ESDB (Commission of the European Communities 1985: http://eusoils.jrc.ec.europa.eu/). The original raster (name PARMADO, resolution 1 × 1 km) was then converted into groups of major geological units (Supplementary Table S3) to standardise any differences in geological unit interpretation between different countries. To account for any possible inaccuracy in the raster on the local scale, the specified geological data was analysed according to our localities, and the pixel assignment was changed in the raster if necessary. Finally, the geology of the localities was assigned based on the converted raster using ArcGIS DesktopTM 9.2: Spatial Analyst tools Extract values to point tool [ESRI Inc. 1999–2008, Redlands, CA, USA] (Supplementary Table S3).

### Gradient analysis

The distribution of the studied cytotypes was then analysed according to climatic and altitudinal gradients using principal component analysis (PCA). In the PCA, the localities were treated as 'cases' and six of the bioclimatic parameters of localities as 'variables'. Unconstrained, linear PCA analysis was performed using Canoco software; the analysis was run together with default centring and standardizing of the general environmental data^65^. Altitude was related to the multidimensional PCA space of the climatic parameters using the Generalized Additive Model. The model parameters were as follows: response variable—altitude, predictors—PCA axes, response distribution—Gaussian, link function—identity. Stepwise selection of models was performed using the AIC (Akaike information criterion) and different Term Smoothness values. The lowest AIC was demonstrated in the model with Term Smoothness = 5.0 for both predictor 1 and predictor 2. Ordination, modelling, and graphic plots were performed using Canoco 5 software^65^.

The analysis of the cytotype occurrences along the altitude gradient, and according to habitat and geological units, was performed using ridgeline plots in the *ggridges* package in R^66^. These are partially overlapping line plots that can be useful for visualizing changes in distributions along environmental gradients.

### Modelling of potentially suitable areas

The predictive modelling of the distribution and niche analysis was performed using Maxent (version: 3.4.0), a machine-learning method which uses environmental variables presented in raster layers to predict the suitability of distributions or habitats^67–69^. Based on the maximum-entropy principle, Maxent finds the most probable distribution for a species that maximizes the entropy^70^.

The raster layers used in the analysis were restricted to the background area; these included the whole range of the studied localities and covered a broad area of Europe. The layer with the parent material of soils was treated as categorical, and the six bioclimatic layers as numerical. Geological data were used to increase the realism and spatial precision of the results, as shown in our previous studies on *F. amethystina*^29,30^. The raster with the parent material of soils was transformed to match cell sizes with the bioclimatic data. After transformation into an ESRI ASCII format, the obtained data set was used in Maxent modelling. All the above transformations and point generations and extractions were done in ArcGIS DesktopTM 9.2: Spatial Analyst tools and Conversion tools [ESRI Inc. 1999–2008, Redlands, CA, USA].

Our models were fitted using hinge features with default regularization parameters. All of the models were fitted on the basis of the full data sets, and tenfold cross-validation was used to estimate errors around the fitted functions and predictive performance of the held-out data^70^.

### Model selection

Because the studied localities are not equally distributed across the study area, the models based on all localities were compared with a model based on spatially filtered data. Spatial filtering is an approved solution when small sample sizes are used with respect to the study area, as demonstrated in a previous study of *F. amethystina*^30^. To select the best model, we used specific evaluation measures, such as AIC (Akaike information criterion), BIC (Bayesian information criterion), and AUC (Area Under the Receiver Operating Characteristics Curve)^69^. AIC and BIC indexes were calculated in the ENMTools software^71^, and AUC was calculated in Maxent. Detailed results of model selection are included in Supplementary Table S4. Generally, models with a filtered sample-set resulted in the best model quality measurements and were used in further analyses.

### Modelling of potential ranges

To determine the influence of differences between niches on potential distribution of cytotypes, the prediction maps were developed. The Maxent prediction map provides a geographical visualization of the prediction model, which can be interpreted as the predicted distribution of the most suitable areas for particular cytotypes^70^.

The potential distribution of suitable areas for both cytotypes was modelled in the current climate, using the geology layer as a predictor. The prediction maps were generated in ASCII file format and were visualized in ArcMap 9.2 software (ESRI Inc. 1999–2008, Redlands, CA, USA).

We generated binary (presence, absence) maps for each cytotype. To obtain binary predictions of the climate + geology suitability of ENMs, MaxEnt’s logistic probability of occurrence output was converted to a binary mode (presence–absence output) using the maximum training sensitivity plus specificity logistic (MTSS) threshold. By maximizing the proportions of actual positives and negatives that were correctly identified, MTSS-based predictions are believed to be the most accurate forecasts of the potential presence or absence of species^72^.

## Supplementary Information

Supplementary Information.
