# Supplementary material for: Tetraploids expanded beyond the mountain niche of their diploid ancestors in the mixed-ploidy grass Festuca amethystina L
Source: Sci Rep. 2021 Sep 21;11:18735. doi: 10.1038/s41598-021-97767-6 (PMC8455632; doi:10.1038/s41598-021-97767-6)
Supplement: Supplementary file 1 — Supplementary Information. [file 41598_2021_97767_MOESM1_ESM.pdf]

Supplementary Materials

Tetraploids expanded beyond the mountain niche of their diploid ancestors in the mixed-ploidy grass Festuca amethystina L.

Marcin Kiedrzyński[1], Katarzyna M. Zielińska[2], Iwona Jedrzejczyk[3], Edyta Kiedrzyńska[4,5], Przemysław P. Tomczyk[1], Agnieszka Rewicz[1], Monika Rewers[3], Adrian Indreica[6], Iryna Bednarska[7], Vladimir Stupar[8], Jan Roleček[9,10], Petr Šmarda[9]

[1] Department of Biogeography, Paleoecology and Nature Conservation, Faculty of Biology and Environmental Protection, University of Lodz, Lodz, Poland

[2] Department of Geobotany and Plant Ecology, Faculty of Biology and Environmental Protection, University of Lodz, Lodz, Poland

[3] Laboratory of Molecular Biology and Cytometry, Department of Agricultural Biotechnology, Bydgoszcz University of Science and Technology, Bydgoszcz, Poland

[4] European Regional Centre for Ecohydrology of the Polish Academy of Sciences, Lodz, Poland

[5] UNESCO Chair on Ecohydrology and Applied Ecology, Faculty of Biology and Environmental Protection, University of Lodz, Lodz, Poland

[6] Department of Silviculture, Transilvania University of Brasov, Brasov, Romania

[7] Department of Nature Ecosystems Protection, Institute of Ecology of the Carpathians NASU, Lviv, Ukraine

[8] Faculty of Forestry, University of Banja Luka, Banja Luka, Bosnia and Herzegovina

[9] Department of Botany and Zoology, Faculty of Science, Masaryk University, Brno, Czech Republic

[10] Department of Paleoecology, Institute of Botany, Czech Academy of Sciences, Brno, Czech Republic

*Corresponding author: marcin.kiedrzynski@biol.uni.lodz.pl (M. Kiedrzyński)

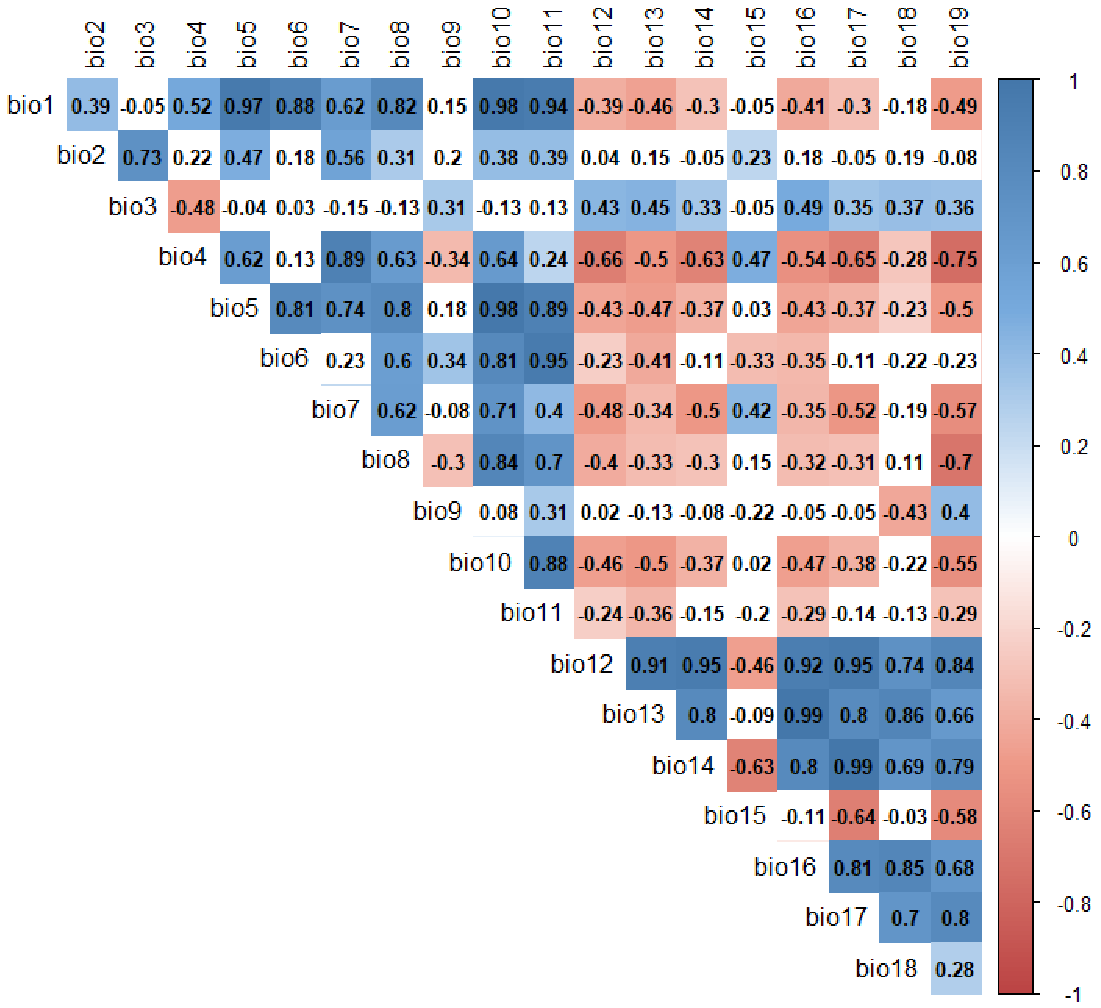

Figure S1. Correlation matrix of bioclimatic values assigned for studied localities of *F. amethystina*. For building the correlation matrix, we used 'corrplot' package in R (Wei and Simko, 2017). We used the default Pearson correlations.

Finally, we chose six variables, within which the three were climatic parameters for temperature and the three for precipitation and included one main parameter and two ancillaries. In the result we used in further analysis the following: Bio_1 – Annual Mean Temperature [oC]; Bio_7 – Annual Temperature Range, Bio_9 – Mean Temperature of the Driest Quarter [oC], Bio_12 – Annual Precipitation [mm]; Bio_18 – Precipitation of the Warmest Quarter [mm] and Bio_19 – Precipitation of the Coldest Quarter [mm].

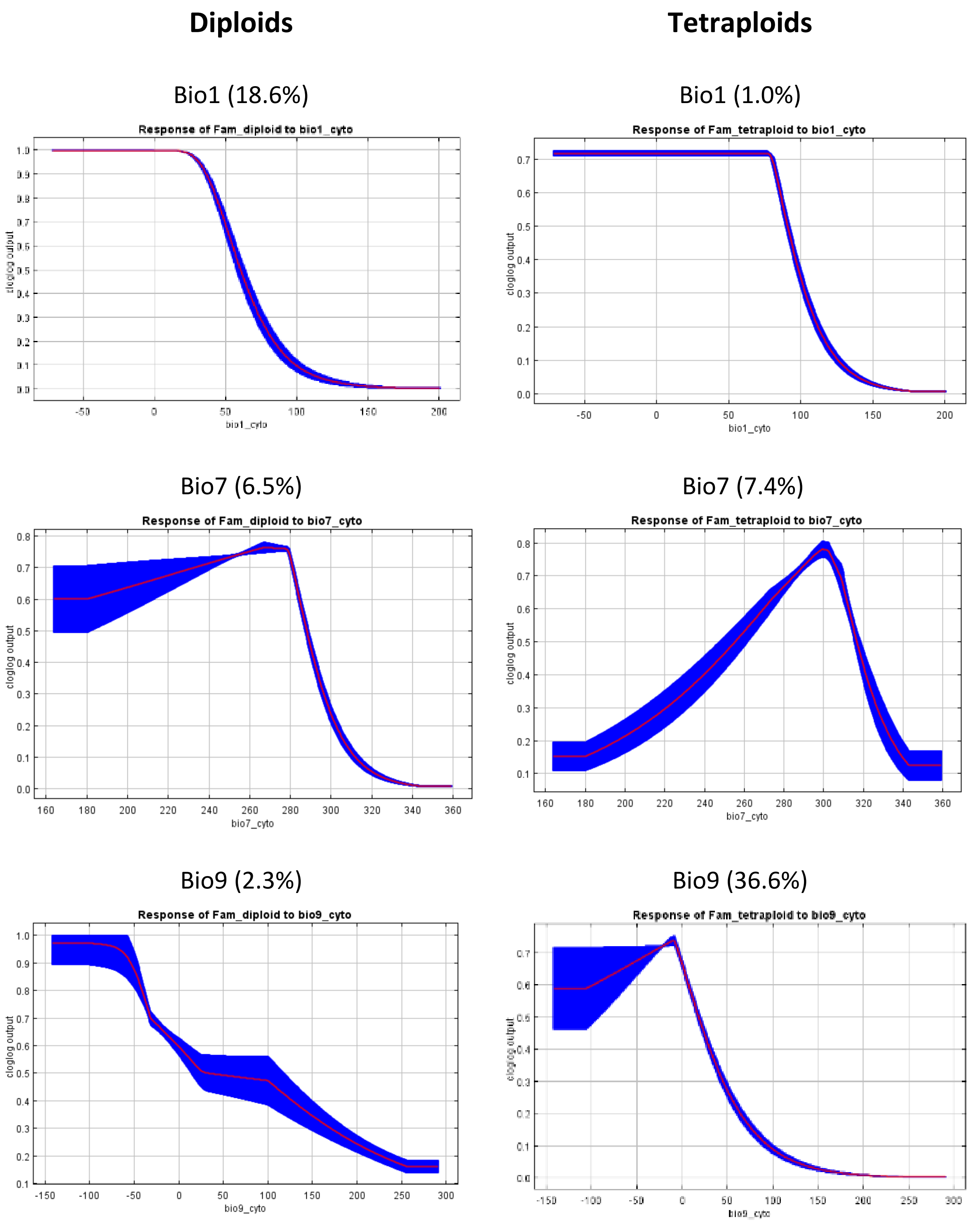

Figure S2. Respond curves of climatic (temperature Bio1, Bio7, Bio9) variables in Maxent models performed for diploids and tetraploids of F. amethystina. Curves indicate the probability of occurrence in a given value of the climatic parameter (scale in °C x 10). Plots are given from models where climatic and geologic predictors were used together (model Clim+Geo). In brackets, the percentage of contribution from Table 1 are given. Red lines on schemes indicate Mean response curves calculated from 10-fold replications, and blue areas indicate Mean +/- Standard Deviation.

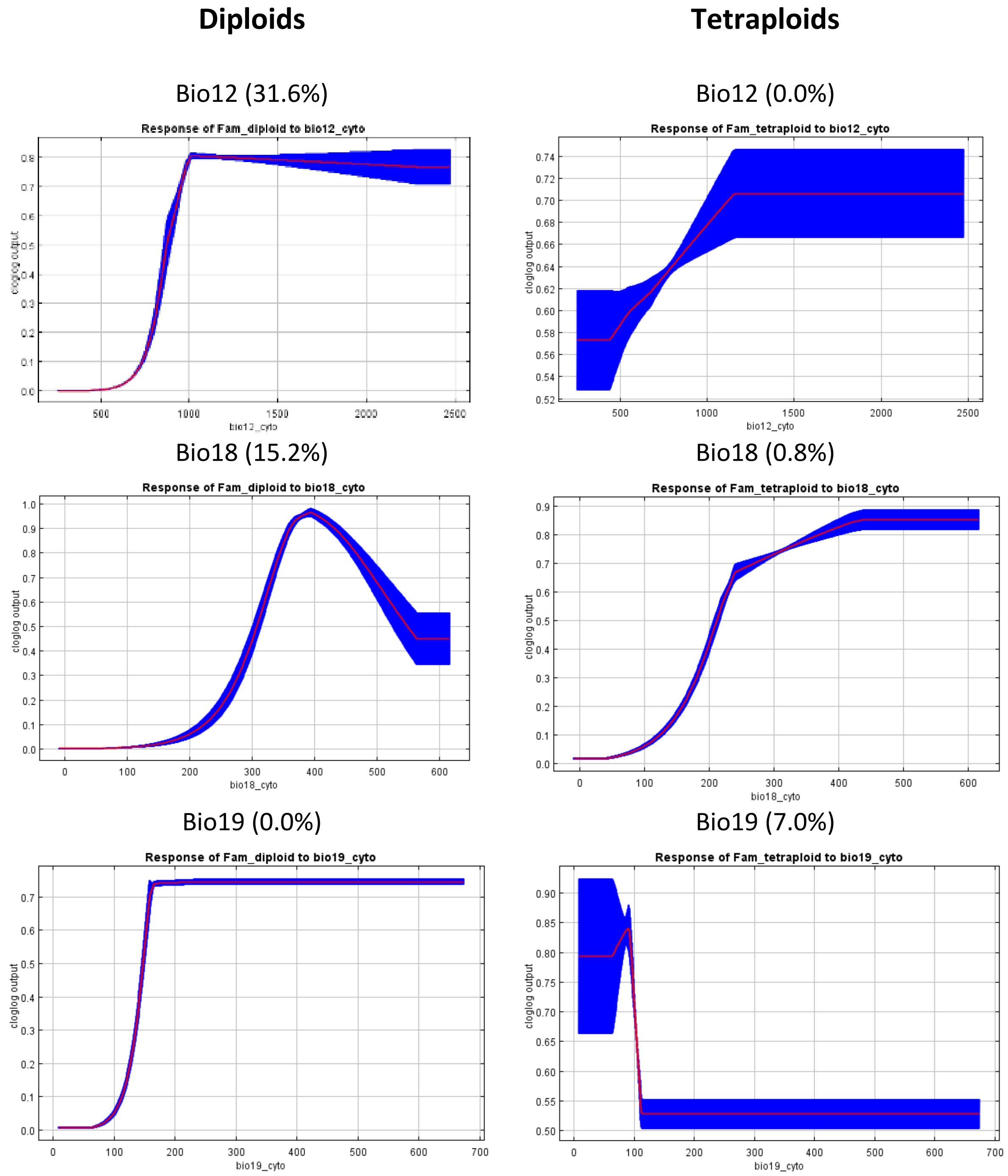

Figure S3. Response curves of climatic (precipitation Bio12, Bio18, Bio19) variables in Maxent models performed for diploids and tetraploids of F. amethystina. Curves indicate the probability of occurrence in a given value of the climatic parameter (scale in mm of precipitation). Plots are given from models where climatic and geologic predictors were used together (model Clim+Geo). In brackets, the percentage of contribution from Table 1 are given. Red lines on schemes indicate Mean response curves calculated from 10-fold replications, and blue areas indicate Mean +/- Standard Deviation.

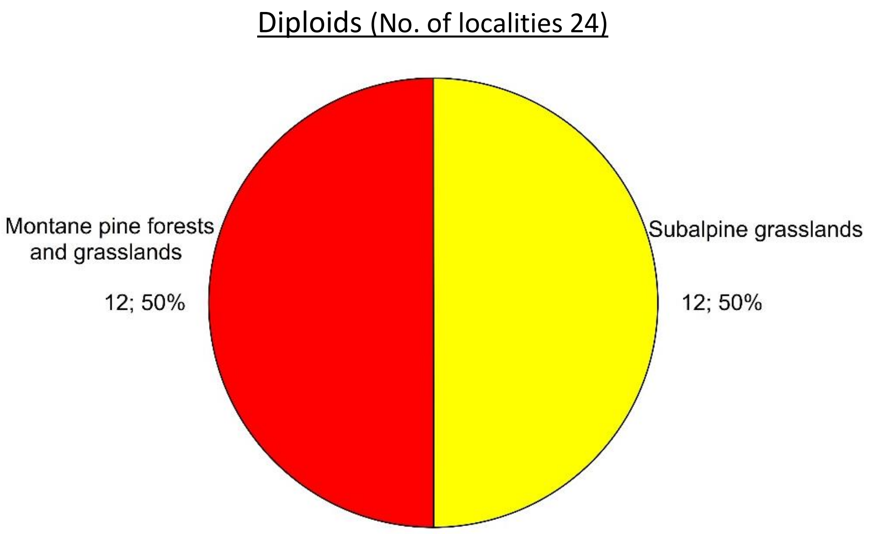

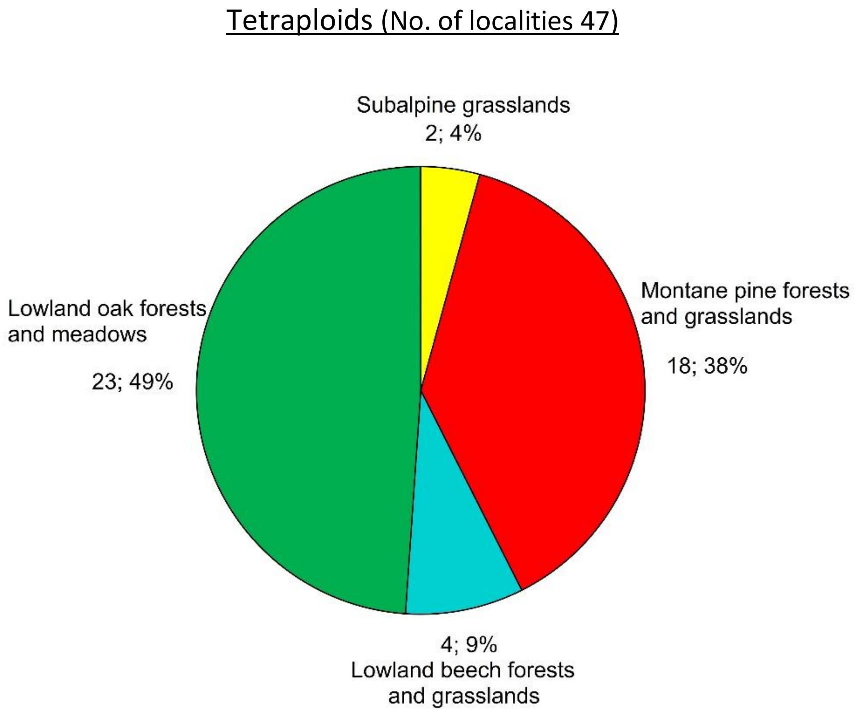

Figure S4. Number of localities and percentage contribution of localities of *Festuca amethystina* cytotypes in types of habitats.

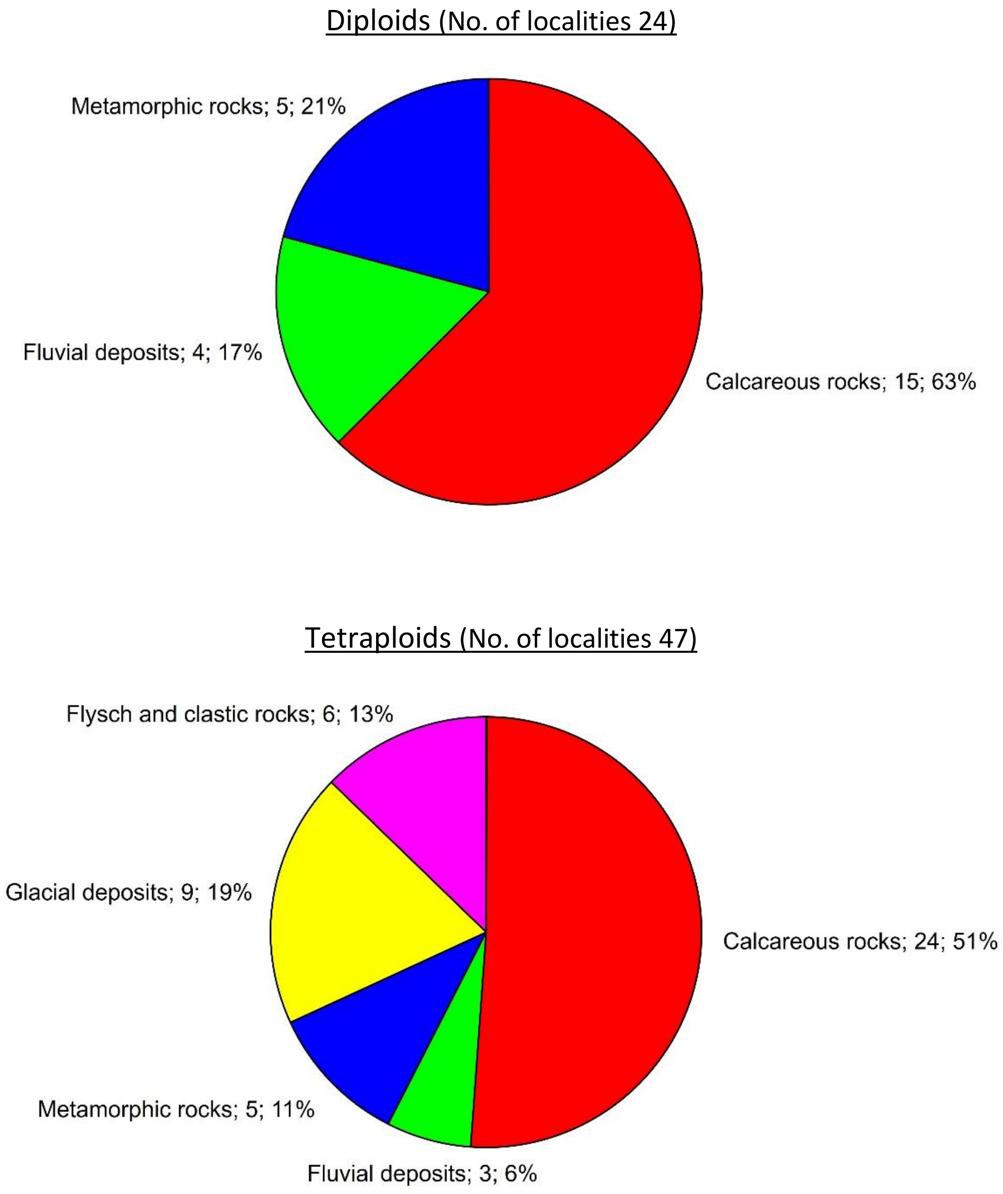

Figure S5. Number of localities and percentage contribution of localities of *Festuca amethystina* cytotypes on types of parent material of soils.

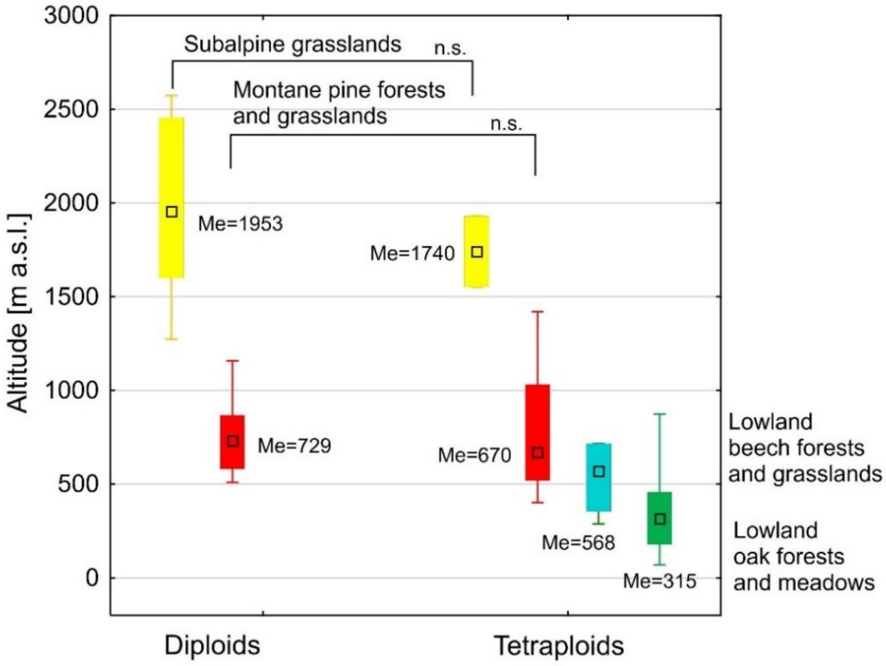

Figure S6. Altitude of localities of *Festuca amethystina* cytotypes according to types of habitats. Boxplots: squares represent the median (Me), the ends of the boxes indicate upper and lower quartiles, the whiskers show the highest and lowest value; n.s. – no significant level of statistical differences between medians according to Mann–Whitney U test.

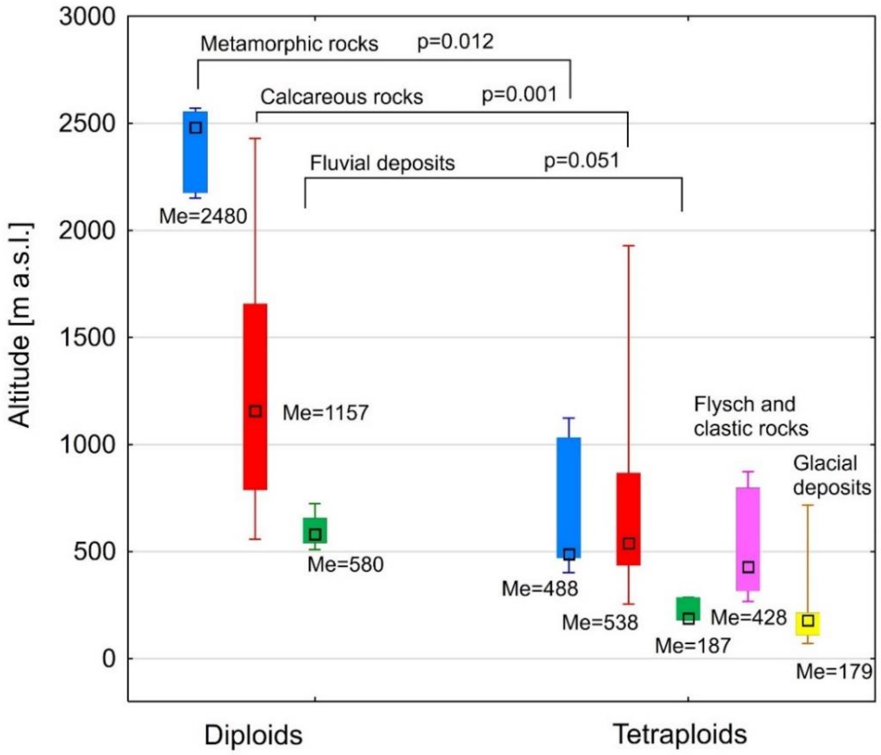

Figure S7. Altitude of localities of *Festuca amethystina* cytotypes according to types of parent material of soils. Boxplots: squares represent the median (Me), the ends of the boxes indicate upper and lower quartiles, the whiskers show the highest and lowest value; p-value – significance level of statistical differences between medians according to Mann–Whitney U test.

Table S1. Ploidy levels of *Festuca amethystina* accessions mesured by flow cytometry.

No.	Locality	Country	No. of samples	Inferred ploidy level	Methodology*	Hebarium voucher stored
**1**	Erl	Austria	8	2x	A	
**2**	Guslica	Croatia	8	2x	A	
**3**	Garmisch-Partenkirchen	Germany	8	2x	A	
**4**	Augsburg	Germany	9	2x	A	
**5**	Grundl	Germany	9	2x	A	
**6**	Wallgau	Germany	9	2x	A	
**7**	Vorderriss	Germany	9	2x	A	
**8**	Caraiman	Romania	8	2x	A	
**9**	Corongis (two subpopulations)	Romania	8	2x	A	
**10**	Capra Valley	Romania	10	2x	A	
**11**	Hasmasul Mare (two subpopulations)	Romania	7	2x	A	
**12**	Suhardul	Romania	8	2x	A	
**13**	Veliko Kozje	Slovenia	8	2x	A	
**14**	Tolsti Vrh	Slovenia	9	2x	A	
**15**	Góra Bliźnica	Ukraine	8	2x	A	
**16**	Góra Dogyaska	Ukraine	8	2x	A	
**17**	Mayrwinkl	Austria	8	4x	A	
**18**	Sankt Martin	Austria	9	4x	A	
**19**	Mattsee	Austria	9	4x	A	
**20**	Strubklamm	Austria	9	4x	A	
**21**	Friesach	Austria	12	4x	A	
**22**	Hubertussee	Austria	9	4x	A	
**23**	Unternberg	Austria	1	4x	A	
**24**	Kreuzbach	Austria	1	4x	A	
**25**	Vlašić	Bosnia and Herzegovina	8	4x	A	
**26**	Buletić	Bosnia and Herzegovina	8	4x	A	
**27**	Klekovača	Bosnia and Herzegovina	10	4x	A	
**28**	Hodonin (two subpopulations)	Czech Republic	6	4x	A	
**29**	Backerbichl	Germany	6	4x	A	
**30**	Hartschimmel	Germany	9	4x	A	
**31**	Bad Reichenhall	Germany	9	4x	A	
**32**	Pilisszentivan	Hungary	4	4x	A	
**33**	Dąbrowa Grotnicka	Poland	8	4x	A	
**34**	Dziki Ostrów	Poland	5	4x	A	
**35**	Grzywy Korzeckowskie	Poland	6	4x	A	
**36**	Jelenia Góra (Sudół)	Poland	20	4x	A	
**37**	Miejskie Pola	Poland	10	4x	A	
**38**	Obrzycko	Poland	5	4x	A	
**39**	Tychów Stary	Poland	4	4x	A	
**40**	Wola Wydrzyna	Poland	11	4x	A	
**41**	Petriceni	Romania	6	4x	A	
**42**	Valea Seaca	Romania	6	4x	A	
**43**	Tornik	Serbia	8	4x	A	
**44**	Bare	Serbia	8	4x	A	
**45**	Borišov	Slovakia	16	4x	A	
**46**	Tlstá	Slovakia	8	4x	A	
**47**	Ostrá	Slovakia	4	4x	A	
**48**	Moasa 1 (NE from Moasa)	Greece	2	2x	B	
**49**	Moasa 2 (S/SE from Moasa)	Greece	2	2x	B	
**50**	Smolikas	Greece	1	2x	B	
**51**	Drahanská vrchovina highlands 1	Czech Republic	10	4x	B	
**52**	Drahanská vrchovina highlands 2	Czech Republic	13	4x	B	
**53**	Houžetín	Czech Republic	6	4x	B	
**54**	Lipou	Czech Republic	6	4x	B	
**55**	Mokrý Vrch - Doutnáč - Velká Hora	Czech Republic	14	4x	B	
**56**	Grunwald	Germany	1	2x	AH	Munich (M)
**57**	Gutmadingen E	Germany	1	2x	AH	Munich (M)
**58**	Tokary-Licheń	Poland	1	4x	AH	Łódź (LOD)
**59**	Zielonka (two subpopulations)	Poland	2	4x	AH	Łódź (LOD)

* A– measured from dry material accordng to Rewicz et al. (2018); AH – measured from herbarium vouchers according to Rewicz et al. (2018); B – measured from fresh material according to Šmarda et al. (2008)

Table S2. Additional localities of Festuca amethystina with known ploidy level

No.	Locality	Country	Source/Reference	Ploidy level
**60**	Golyam Mechi	Bulgaria	Kuzmanov, B., 1993. Chromosome numbers of bulgarian angiosperms : An introduction to a chromosome atlas of the Bulgarian flora. Flora Mediterr. 3, 19–163; F. amethystina 2n=14, according to Petrova A., Kozuharov, S., 1987. Citotaxonomicno proucvane na balgarski vidove ot roda Festuca L. [in:] Trudova IV. Nacionalna Konferencija Po Botanika, 1, 16-23; locality of vegetation patches according to Managment plan of the Rila Monastery Rezerve 2004	2x
**61**	Gutmadingen W	Germany	Gregor T. and Paule J. 2014; according to Paule J., Gregor T., Schmidt M., Gerstner E-M., Dersch G., Dressler S., Wesche K., Zizka G. 2017. Chromosome numbers of the flora of Germany – a new online database of georeferenced chromosome counts and flow cytometric ploidy estimates. Plant Syst. Evol. 303(8):1123-1129.	2x
**62**	Wolfratshausen	Germany	Gregor T. and Paule J. 2014; according to Paule J., Gregor T., Schmidt M., Gerstner E-M., Dersch G., Dressler S., Wesche K., Zizka G. 2017. Chromosome numbers of the flora of Germany – a new online database of georeferenced chromosome counts and flow cytometric ploidy estimates. Plant Syst. Evol. 303(8):1123-1129.	2x
**63**	Mutěnice (near Hodonín)	Czech Republic	Šmarda P. and Kočí K. 2003: Chromosome number variability in central european members of the Festuca ovina and F. pallens groups (sect. Festuca). Folia Geobot. 38/1: 65-95.	4x
**64**	Emmerberg	Austria	Šmarda P. 2008. DNA ploidy level variability of some fescues (Festuca subg. Festuca, Poaceae) from Central and Southern Europe measured in fresh plants and herbarium specimens. Biologia 63(3): 349–367.	4x
**65**	Salzburg-Kuhberg	Austria	Wittmann H. and Strobl W. 1984. Beitrag zur Kenntnis von Festuca amethystina L. im Bundesland Salzburg. Florist. Mitt. Salzburg 9: 3-8.	4x
**66**	Wolfschwang	Austria	Wittmann H. and Strobl W. 1984. Beitrag zur Kenntnis von Festuca amethystina L. im Bundesland Salzburg. Florist. Mitt. Salzburg 9: 3-8.	4x
**67**	Erlangen	Germany	Paule J., Gregor T., Schmidt M., Gerstner E-M., Dersch G., Dressler S., Wesche K., Zizka G. 2017. Chromosome numbers of the flora of Germany – a new online database of georeferenced chromosome counts and flow cytometric ploidy estimates. Plant Systematics and Evolution 303(8):1123-1129.	4x
**68**	Veldensteiner Forst	Germany	Hand R. and Gregor T. 2011. Chromosomenzahlen von Farn- und Samenpflanzen aus Deutschland 5. Kochia 5:33-38.	4x
**69**	Büdöskút	Hungary	Baksay L. 1956, Budapest, Büdöskút, according to Baksay L. 1956. Cytotaxonomical Studies on the Flora of Hungary. Budapest.	4x
**70**	Păucea	Romania	Roleček, J., Dřevojan, P., Šmarda, P., 2019. First record of festuca amethystina L. From the transylvanian basin (Romania). Contrib. Bot. 54: 91–97. https://doi.org/10.24193/Contrib.Bot.54.6	4x
**71**	Králova Skala	Slovakia	Uhríková A. and Bernátová D. 2000: Karyologické štúdium slovenskej flóry XXXII. Acta Fac. Rerum Nat. Univ. Comen. Bot. 40: 31-36.	4x

Table. S3. Localities of analysed F. amethystina samples and their characteristics. Localities numbers correspond to Tables S1 and S2.

No.	Ploidy level	Locality name	Country	Latitude	Longitude	Region	Altitude [m]	Geology	Habitat
**1**	2x	Erl	Austria	47.687	12.182	North-Eastern Alps	557	Calcareous rocks	Montane pine forests and grasslands
**2**	2x	Guslica	Croatia	45.455	14.572	Dinaric Mountains	1439	Calcareous rocks	Subalpine grasslands
**3**	2x	Garmisch-Partenkirchen	Germany	47.489	10.969	North-Eastern Alps	991	Calcareous rocks	Montane pine forests and grasslands
**4**	2x	Augsburg	Germany	48.289	10.927	Alps Foreland	509	Fluvial deposits	Montane pine forests and grasslands
**5**	2x	Grundl	Germany	47.702	10.803	Alps Foreland	724	Fluvial deposits	Montane pine forests and grasslands
**6**	2x	Wallgau	Germany	47.522	11.278	North-Eastern Alps	941	Calcareous rocks	Montane pine forests and grasslands
**7**	2x	Vorderriss	Germany	47.553	11.414	North-Eastern Alps	793	Calcareous rocks	Montane pine forests and grasslands
**8**	2x	Moasa 1 (NE from Moasa)	Greece	40.080	20.951	Pindus Mountains	2480	Metamorphic rocks	Subalpine grasslands
**9**	2x	Moasa 2 (S/SE from Moasa)	Greece	40.089	20.959	Pindus Mountains	2177	Metamorphic rocks	Subalpine grasslands
**10**	2x	Smolikas	Greece	40.089	20.924	Pindus Mountains	2555	Metamorphic rocks	Subalpine grasslands
**11**	2x	Caraiman	Romania	45.426	25.481	Eastern and Southern Carpathians	2429	Calcareous rocks	Subalpine grasslands
**12**	2x	Corongis (two subpopulations)	Romania	47.530	24.797	Eastern and Southern Carpathians	1756	Calcareous rocks	Subalpine grasslands
**13**	2x	Capra Valley	Romania	45.596	24.635	Eastern and Southern Carpathians	2152	Metamorphic rocks	Subalpine grasslands
**14**	2x	Hasmasul Mare (two subpopulations)	Romania	46.702	25.808	Eastern and Southern Carpathians	1688	Calcareous rocks	Subalpine grasslands
**15**	2x	Suhardul	Romania	46.799	25.798	Eastern and Southern Carpathians	1157	Calcareous rocks	Montane pine forests and grasslands
**16**	2x	Veliko Kozje	Slovenia	46.080	15.226	Southern Alps	788	Calcareous rocks	Montane pine forests and grasslands
**17**	2x	Tolsti Vrh	Slovenia	46.344	14.358	Southern Alps	1273	Calcareous rocks	Subalpine grasslands
**18**	2x	Góra Bliźnica	Ukraine	48.264	24.209	Eastern and Southern Carpathians	1539	Calcareous rocks	Subalpine grasslands
**19**	2x	Góra Dogyaska	Ukraine	48.271	24.162	Eastern and Southern Carpathians	1657	Calcareous rocks	Subalpine grasslands
**20**	4x	Mayrwinkl	Austria	47.743	14.319	North-Eastern Alps	667	Calcareous rocks	Montane pine forests and grasslands
**21**	4x	Sankt Martin	Austria	47.544	12.719	North-Eastern Alps	662	Calcareous rocks	Montane pine forests and grasslands
**22**	4x	Mattsee	Austria	47.970	13.095	Alps Foreland	520	Calcareous rocks	Montane pine forests and grasslands
**23**	4x	Strubklamm	Austria	47.775	13.198	North-Eastern Alps	725	Calcareous rocks	Montane pine forests and grasslands
**24**	4x	Friesach	Austria	47.175	15.344	North-Eastern Alps	479	Calcareous rocks	Montane pine forests and grasslands
**25**	4x	Hubertussee	Austria	47.811	15.371	North-Eastern Alps	849	Calcareous rocks	Montane pine forests and grasslands
**26**	4x	Unternberg	Austria	48.075	15.142	Alps Foreland	287	Fluvial deposits	Lowland beech forests and meadows
**27**	4x	Kreuzbach	Austria	47.867	15.995	North-Eastern Alps	459	Calcareous rocks	Montane pine forests and grasslands
**28**	4x	Vlašić	Bosnia and Herzegovina	44.277	17.613	Dinaric Mountains	1552	Calcareous rocks	Subalpine grasslands
**29**	4x	Buletić	Bosnia and Herzegovina	44.572	17.729	Dinaric Mountains	402	Metamorphic rocks	Montane pine forests and grasslands
**30**	4x	Klekovača	Bosnia and Herzegovina	44.431	16.509	Dinaric Mountains	1929	Calcareous rocks	Subalpine grasslands
**31**	4x	Drahanská vrchovina highlands 1	Czech Republic	NA	NA	Bohemian Massiv and Franconian Jura	471	Metamorphic rocks	Lowland oak forests and meadows
**32**	4x	Drahanská vrchovina highlands 2	Czech Republic	NA	NA	Bohemian Massiv and Franconian Jura	488	Metamorphic rocks	Lowland oak forests and meadows
**33**	4x	Houžetín	Czech Republic	50.489	13.883	Bohemian Massiv and Franconian Jura	499	Calcareous rocks	Lowland oak forests and meadows
**34**	4x	Lipou	Czech Republic	49.942	14.104	Bohemian Massiv and Franconian Jura	356	Calcareous rocks	Lowland oak forests and meadows
**35**	4x	Mokrý Vrch - Doutnáč - Velká Hora	Czech Republic	49.956	14.158	Bohemian Massiv and Franconian Jura	359	Calcareous rocks	Lowland oak forests and meadows
**36**	4x	Hodonín (two subpopulations)	Czech Republic	48.876	17.103	Bohemian Massiv and Franconian Jura	188	Fluvial deposits	Lowland oak forests and meadows

Table. S3. …Continuation

**37**	4x	Backerbichl	Germany	47.966	11.204	Alps Foreland	717	Glacial deposits	Lowland beech forests and meadows
**38**	4x	Hartschimmel	Germany	47.933	11.186	Alps Foreland	717	Glacial deposits	Lowland beech forests and meadows
**39**	4x	Bad Reichenhall	Germany	47.705	12.859	North-Eastern Alps	526	Calcareous rocks	Montane pine forests and grasslands
**40**	4x	Pilisszentivan	Hungary	47.568	18.879	Transdanubian Mountains	420	Calcareous rocks	Lowland beech forests and meadows
**41**	4x	Dąbrowa Grotnicka	Poland	51.917	19.319	Polish Uplands and Lowlands	179	Glacial deposits	Lowland oak forests and meadows
**42**	4x	Dziki Ostrów	Poland	53.012	18.018	Polish Uplands and Lowlands	85	Glacial deposits	Lowland oak forests and meadows
**43**	4x	Grzywy Korzeckowskie	Poland	50.796	20.400	Polish Uplands and Lowlands	331	Calcareous rocks	Lowland oak forests and meadows
**44**	4x	Jelenia Góra (Sudół)	Poland	50.979	21.488	Polish Uplands and Lowlands	214	Glacial deposits	Lowland oak forests and meadows
**45**	4x	Miejskie Pola	Poland	51.070	19.929	Polish Uplands and Lowlands	255	Calcareous rocks	Lowland oak forests and meadows
**46**	4x	Obrzycko	Poland	52.694	16.581	Polish Uplands and Lowlands	70	Glacial deposits	Lowland oak forests and meadows
**47**	4x	Tychów Stary	Poland	51.102	21.080	Polish Uplands and Lowlands	266	Flysch and clastic rocks	Lowland oak forests and meadows
**48**	4x	Wola Wydrzyna	Poland	51.248	19.187	Polish Uplands and Lowlands	192	Glacial deposits	Lowland oak forests and meadows
**49**	4x	Petriceni	Romania	46.081	26.090	Eastern and Southern Carpathians	873	Flysch and clastic rocks	Lowland oak forests and meadows
**50**	4x	Valea Seaca	Romania	46.135	26.118	Eastern and Southern Carpathians	798	Flysch and clastic rocks	Lowland oak forests and meadows
**51**	4x	Tornik	Serbia	43.670	19.643	Dinaric Mountains	1124	Metamorphic rocks	Montane pine forests and grasslands
**52**	4x	Bare	Serbia	43.894	19.560	Dinaric Mountains	1033	Metamorphic rocks	Montane pine forests and grasslands
**53**	4x	Borišov	Slovakia	48.941	19.093	Western Carpathians	1420	Calcareous rocks	Montane pine forests and grasslands
**54**	4x	Tlstá	Slovakia	48.932	18.975	Western Carpathians	1331	Calcareous rocks	Montane pine forests and grasslands
**55**	4x	Ostrá	Slovakia	48.906	18.962	Western Carpathians	673	Calcareous rocks	Montane pine forests and grasslands
**56**	2x	Grunwald	Germany	48.046	11.536	Alps Foreland	591	Fluvial deposits	Montane pine forests and grasslands
**57**	2x	Gutmadingen E	Germany	47.911	8.628	Swabian Jura	736	Calcareous rocks	Montane pine forests and grasslands
**58**	4x	Tokary-Licheń	Poland	52.353	18.403	Polish Uplands and Lowlands	111	Glacial deposits	Lowland oak forests and meadows
**59**	4x	Zielonka (two subpopulations)	Poland	52.543	17.137	Polish Uplands and Lowlands	109	Glacial deposits	Lowland oak forests and meadows
**60**	2x	Golyam Mechi	Bulgaria	42.060	23.439	Rila Mountains	2572	Metamorphic rocks	Subalpine grasslands
**61**	2x	Gutmadingen W	Germany	47.911	8.614	Swabian Jura	676	Calcareous rocks	Montane pine forests and grasslands
**62**	2x	Wolfratshausen	Germany	47.937	11.437	Alps Foreland	571	Fluvial deposits	Montane pine forests and grasslands
**63**	4x	Mutěnice (near Hodonín)	Czech Republic	48.886	17.077	Bohemian Massiv and Franconian Jura	180	Fluvial deposits	Lowland oak forests and meadows
**64**	4x	Emmerberg	Austria	47.819	16.102	North-Eastern Alps	448	Calcareous rocks	Montane pine forests and grasslands
**65**	4x	Salzburg-Kuhberg	Austria	47.810	13.076	North-Eastern Alps	552	Calcareous rocks	Montane pine forests and grasslands
**66**	4x	Wolfschwang	Austria	47.714	12.934	North-Eastern Alps	884	Calcareous rocks	Montane pine forests and grasslands
**67**	4x	Erlangen	Germany	49.539	11.036	Bohemian Massiv and Franconian Jura	316	Flysch and clastic rocks	Lowland oak forests and meadows
**68**	4x	Veldensteiner Forst	Germany	49.675	11.542	Bohemian Massiv and Franconian Jura	458	Flysch and clastic rocks	Lowland oak forests and meadows
**69**	4x	Büdöskút	Hungary	46.810	17.328	Transdanubian Mountains	375	Calcareous rocks	Lowland oak forests and meadows
**70**	4x	Păucea	Romania	46.226	24.340	Eastern and Southern Carpathians	399	Flysch and clastic rocks	Lowland oak forests and meadows
**71**	4x	Králova Skala	Slovakia	48.886	19.046	Western Carpathians	1337	Calcareous rocks	Montane pine forests and grasslands

Table S4. Model selection in Maxent modeling of potential ranges in F. amethystina polyploid complex. Bold values indicated significantly better models, according to models quality indexes: AICc and BIC. Models' performance, according to Maxent AUC values, does not differ in compared models. AIC and BIC indexes were calculated in the ENMTools software (Warren et al., 2010), and AUC was calculated in Maxent software (Phillips et al., 2006, 2017; Warren and Seifert, 2011).

Predictors	Cytotype	Sample size	AICc			BIC			Maxent AUC	
			Mean	*Min*	*Max*	*Mean*	*Min*	*Max*	*Mean*	*Model performance*
only climatic	F. amethystina2x	filtered	**463**	453	468	**455**	451	460	0.950	excellent
		full	1131	780	1891	710	702	721	0.961	excellent
	F. amethystina4x	filtered	**993**	980	1005	**995**	986	1003	0.725	moderate
		full	1407	1399	1422	1420	1412	1432	0.805	moderate
climatic and edaphic	F. amethystina2x	filtered	**461**	445	498	**438**	437	441	0.974	excellent
		full	1462	1200	1859	680	677	689	0.979	excellent
	F. amethystina4x	filtered	**1003**	977	1036	**985**	975	996	0.803	moderate
		full	1385	1365	1402	1392	1378	1403	0.845	moderate

Table S5. Potential contact zones between populations of cytotypes (diploids and tetraploids) of *Festuca amethystina*.

No. according to Figure 2	Location	Characteristics	The nearest currently identified distance between locations of diploid and tetraploid populations
1.	The northern Alpine forelands	Diploids grow in pre-alpine river valleys in pine forests; tetraploids on hills in beech forests and meadows.	20 km
2.	The north part of the Eastern Alps	Both cytotypes grow in medium-montane pine forests.	40 km
3.	The south part of the Eastern Alps vs. the Julian Alps	Tetraploids grow in medium-montane pine forests in the south part of the Eastern Alps; diploids grow in medium-montane pine forests and grasslands in the Julian Alps.	100 km
4.	The northern part of the Dinaric Mts.	Diploids grow in the northern part of the region in subalpine grasslands; tetraploids grow in the central parts of the region in medium-montane pine forests and subalpine grasslands.	120-150 km
5.	The southern part of the Dinaric Mts. vs. Pindus Mts. and Rila Mts.	Tetraploids grow in medium-montane pine forests and grasslands in the Dinaric Mts.; diploids grow in subalpine grasslands in the Pindus Mts. and the Rila Mts.	150-300 km
6.	The Eastern Carpathians and the Southern Carpathians vs. the Inner-Eastern Carpathians and the Transylvanian Plateau	Diploids grow in medium-montane pine forests and subalpine grasslands in the Eastern and Southern Carpathians; tetraploids grow in oak forests and meadows in the Inner-Eastern Carpathians and the Transylvanian Plateau.	50-70 km

References

Phillips, S.J., Anderson, R.P., Schapire, R.E. Maximum entropy modeling of species geographic distributions. *Ecol. Modell.* **190**, 231–259. (2006) https://doi.org/10.1016/j.ecolmodel.2005.03.026

Phillips, S.J., Anderson, R.P., Dudík, M., Schapire, R.E., Blair, M.E. Opening the black box: an open-source release of Maxent. *Ecography.* **40**, 887–893. (2017) https://doi.org/10.1111/ecog.03049

Rewicz, A. *et al.* Morphometric traits in the fine-leaved fescues depend on ploidy level: The case of *Festuca amethystina* L. *PeerJ.* **2018**, e5576 (2018).

Šmarda, P., Bureš, P., Horová, L., Foggi, B. & Rossi, G. Genome size and GC content evolution of *Festuca*: Ancestral expansion and subsequent reduction. *Ann. Bot.* **101**, 421–433 (2008).

Warren, D.L., Glor, R.E., Turelli, M. ENMTools: A toolbox for comparative studies of environmental niche models. *Ecography*. **33**, 607–611. (2010) https://doi.org/10.1111/j.1600-0587.2009.06142.x

Warren, D.L., Seifert, S. Ecological niche modeling in Maxent: the importance of model complexity and the performance of model selection criteria. *Ecol. Soc. Am.* **21**, 335–342. (2011) https://doi.org/10.1890/10-1171.1

Wei, T., Simko, V. R package "corrplot": Visualization of a Correlation Matrix (Version 0.84). (2017) Available from https://github.com/taiyun/corrplot
